# Muscular Swedish mutant APP-to-Brain axis in the development of Alzheimer’s disease

**DOI:** 10.1038/s41419-022-05378-4

**Published:** 2022-11-10

**Authors:** Jin-Xiu Pan, Daehoon Lee, Dong Sun, Kai Zhao, Lei Xiong, Hao-Han Guo, Xiao Ren, Peng Chen, Raquel Lopez de Boer, Yuyi Lu, Helena Lin, Lin Mei, Wen-Cheng Xiong

**Affiliations:** 1 Department of Neurosciences, School of Medicine, Case Western Reserve University, Cleveland, OH USA; 2 Louis Stokes Cleveland Veterans Affairs Medical Center, Cleveland, OH USA

**Keywords:** Alzheimer's disease, Neurodegenerative diseases

## Abstract

Alzheimer’s disease (AD) is the most common form of dementia. Notably, patients with AD often suffer from severe sarcopenia. However, their direct link and relationship remain poorly understood. Here, we generated a mouse line, *TgAPP*_*swe*_^*HSA*^, by crossing *LSL (LoxP-STOP-LoxP)-APP*_*swe*_ with *HSA-Cre* mice, which express APP_swe_ (Swedish mutant APP) selectively in skeletal muscles. Examining phenotypes in *TgAPP*_*swe*_^*HSA*^ mice showed not only sarcopenia-like deficit, but also AD-relevant hippocampal inflammation, impairments in adult hippocampal neurogenesis and blood brain barrier (BBB), and depression-like behaviors. Further studies suggest that APP_swe_ expression in skeletal muscles induces senescence and expressions of senescence-associated secretory phenotypes (SASPs), which include inflammatory cytokines and chemokines; but decreases growth factors, such as PDGF-BB and BDNF. These changes likely contribute to the systemic and hippocampal inflammation, deficits in neurogenesis and BBB, and depression-like behaviors, revealing a link of sarcopenia with AD, and uncovering an axis of muscular APP_swe_ to brain in AD development.

## Introduction

Alzheimer’s disease (AD) is the most common form of dementia. Pathologically, it is characterized by cortical and cerebrovascular β-amyloid (Aβ) plaques, phospho-tau containing neurofibrillary tangles, reactive glial cell-associated chronic inflammation, and neuronal loss [1, 2]. Interestingly, in addition to the brain pathology, AD patients often have sarcopenia, a condition characterized by the loss of skeletal muscle mass and function. Clinically, the sarcopenia appears to be tightly associated with the dementia and AD progression, correlating well with the severity of AD [3, 4]. A significant higher prevalence rate of sarcopenia in AD (early to moderate) patients than that of same-aged population with normal cognition has been reported; [3–5] and the poor muscle functions or lower muscle mass in patients with sarcopenia have been implicated as a driver for their association with later-life cognitive impairment [6, 7]. Etiologically, both AD and sarcopenia disorders share several common environmental risk factors, including aging and chronic inflammation, and a few genetic risk genes, such as *ApoE* [7–14]. While these clinical, genetic, and epidemic studies indicate a strong association of AD with sarcopenia, it remains possible that their association is a random coincidence due to their shared environmental risk factors, and it remains unclear exactly how they are linked and what are their relationship(s) are.

We chose Swedish mutant APP (APP_swe_) to address above questions for the following reasons. First, APP_swe_ is one of the earliest mutants identified in patients with early-onset AD (EOAD). Although APP_swe_ is detected in small fractions of EOAD patients, its functions in Aβ production in the brain and in promoting AD pathogenesis have been well studied in multiple animal models (e.g., Tg2576 and 5XFAD, both well-characterized AD animal models that express *APP*_*swe*_ under the control of prion and *Thy1* promoter, respectively). Second, much AD research has been focused on the impact of Aβ on the brain, even though *App* or *APP*_*swe*_ is known to be expressed not only in the brain, but also in periphery tissues [15, 16], including skeletal muscles [17]. While investigating phenotypes in APP_swe_-based animal models have provided valuable insights into Aβ pathology in the brain and impairments in mouse cognitive functions, the functions of APP_swe_ in periphery tissues, such as muscles, remain poorly understood. Third, APP’s physiological function in muscles has been emerged. In addition to its age-dependent expression in muscles and NMJs (neuro-muscular junction) [17], mice with APP knocking out show dysfunctional NMJs with aberrant localization of presynaptic proteins, reduced synaptic vesicles, and abnormal postsynaptic AChR clusters [18]. Fourth, altered expression or increased cleavage of APP appears to be involved in multiple types of human muscle degenerative diseases (see Supplemental Table 1). For examples, muscle fibers of patients with inclusion-body myositis (IBM) have intra-fiber “plaque-like” Aβ accumulation [19], which are believed to promote myofiber degeneration, atrophy, and death [20]. Aβ accumulation in strophic muscles fibers is a key factor in GNE myopathy [21]. Aβ accumulation is also detected in muscles of patients with ALS (amyotrophic lateral sclerosis) and ALS mouse models [22]. In AD patients, Aβ levels are elevated not only in the brain, but also in muscles (e.g., temporalis) [23]. Finally, examinations of muscle structures in *Tg2576*, the well-characterized AD animal model that expresses APP_swe_ ubiquitously, have revealed early-onset sarcopenia-like deficits, months before any brain-pathologic defect that can be detected [24, 25]. Taken together, these observations argue against the view for sarcopenia-like deficits as a consequence of neurodegeneration, or a random coincidence, implicate dysfunctional APP or Aβ as a potential common denominator for AD and muscle degenerative diseases, and raise additional question- could problems in muscles contribute to AD pathology in the brain?

Here, we addressed this question by use of a newly generated APP_swe_-based animal model, *TgAPP*_*swe*_^*HSA*^ mice, which express APP_swe_ specifically in muscles. Investigating phenotypes in *TgAPP*_*swe*_^*HSA*^ mice showed not only earlier onset sarcopenia-like muscle deficits, but also age-dependent depression-like behaviors and brain pathology (largely in the hippocampus), such as increased glial activation, impaired BBB, and elevated pro-inflammatory cytokines. Further studies demonstrate increased senescence and SASPs in APP_swe_ expressing muscles or C2C12 cells; and inhibition of senescence by its inhibitors diminishes or abolishes nearly all the phenotypes in *TgAPP*_*swe*_^*HSA*^ mice. These results thus demonstrate a contribution of muscular APP_swe_ to the development of both AD and sarcopenia, revealing a link between sarcopenia and AD, and uncovering a muscle-to-brain crosstalk for AD development.

## Materials and methods

The *LSL-APP*_*swe*_ mice were generated using the pCCALL2 plasmid as described previously [26]. *TgAPP*_*swe*_^*HSA*^ mice were generated by crossing *LSL-APP*_*swe*_ with *HSA-Cre* mice (purchased from Jackson laboratory, which is donated by Dr. IMR Colony, stock #006149) [27]. This study was conducted in accordance with the National Institutes of Health (NIH) guidelines and the Institutional Animal Care and Use Committee at Case Western Reserve University approved protocols (IACUC, 2017–0121 and 2017-0115).

The other material and methods including Animals, Reagents, Behavioral tests, Stereological cell counting, Histologic staining [H&E, NADH-TR (transferase), SDH (succinate dehydrogenase), COX (Cytochrome c oxidase), PAS (Periodic Acid Schiff), and Gomori-trichrome], Immunofluorescence staining and image analysis, Western blotting, EdU injection and labeling, L-Series label-multiplex antibody arrays, SA-β-gal staining, Elisa assays, RNA isolation, and RT-qPCR were described in supplemental information.

## Results

### *TgAPP*_*swe*_^*HSA*^, a mouse model that selectively expressing APP_swe_ in skeletal muscles

To investigate possible skeletal muscular APP_swe_’s effect in AD and sarcopenia development, we generated *TgAPP*_*swe*_^*HSA*^ or *LSL-APP*_*swe*_*:HSA-Cre* mice by crossing *LSL(Loxp-Stop-Loxp)-hAPP*_*swe*_ (human APP_swe_) with *HSA-Cre (human skeletal a-actin promoter driven Cre)* mice (Fig. S1A). The *APP*_*swe*_ expression in *TgAPP*_*swe*_^*HSA*^ mice is thus under the control of both the CAG promotor (a chicken β-actin promotor with a CMV enhancer to express its mRNAs) and the HSA-Cre dependent removal of LSL in *LSL-hAPP*_*swe*_ mice (Fig. S1A) [26]. The specific expression of the HSA-Cre activity in the skeletal muscles was verified in *HSA-*Cre: *Ai9* mice (where the tdTomato expression depends on Cre activity) (Fig. S1B), in line with literature reports [27]. We further examined *hAPP*_*swe*_’s expression in *TgAPP*_*swe*_^*HSA*^ mice. RT-qPCR analysis using specific primers for human *APP* detected *hAPP*’s transcripts only in skeletal muscles, but un-detectable in the brain-hippocampus or cortex, nor other tissues/organs (such as heart, lung, liver, and kidney) of *TgAPP*_*swe*_^*HSA*^ mice (Fig. S1C). Among different muscles, the *hAPP*_*swe*_’s transcripts were abundantly expressed in tibialis anterior (TA) (a distal fast twitch type), Quadriceps (a proximal fast twitch type), and soleus (a slow twitch type) in *TgAPP*_*swe*_^*HSA*^ *mice* (Fig. S1C). Western blot analysis also showed selectively expression of hAPP_swe_ protein in TA muscles, but not in the cortex nor hippocampus in *TgAPP*_*swe*_^*HSA*^ mice (Fig. S1D, E), verified the RT-qPCR results.

### Decreased muscle fiber size and increased central nuclei in *TgAPP*_*swe*_^*HSA*^ mice in muscle fiber type- and age-dependent manners

**Fig. 1:**
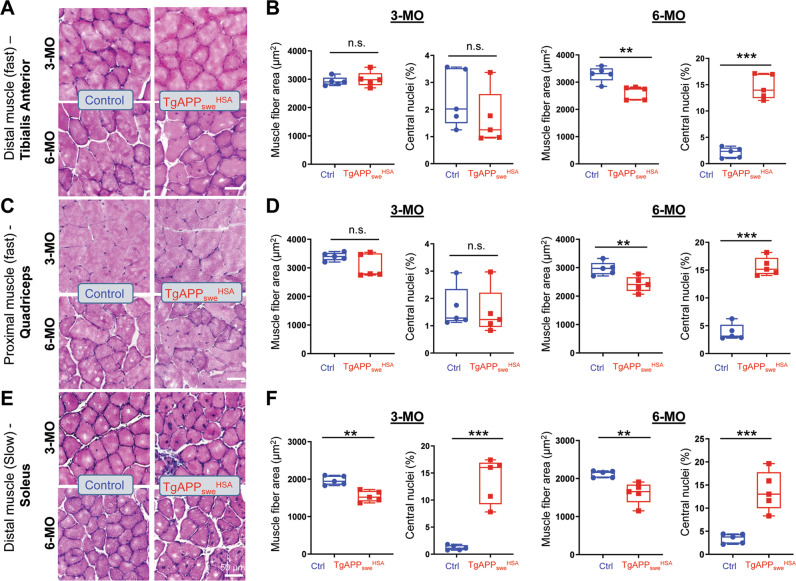
Decreased muscle fiber size and increased central nuclei in *TgAPP*_*swe*_^*HSA*^ mice in muscle fiber type- and age-dependent manners. H & E staining analysis of indicated muscle fibers from 3- and 6-MO control and *TgAPP*_*swe*_^*HSA*^ mice. **A**, **C**, **E** Representative images of TA (distal fast-twitch muscle), quadriceps (proximal fast-twitch muscle), and soleus (slow-twitch muscle), from control and *TgAPP*_***swe***_^***HSA***^ mice at the indicated age, respectively, Scale bar 50 µm. **B**, **D**, **F** Quantification analyses of mean muscle fiber size area and percentage of muscles with central nuclei in indicated mice at 3- and 6-MO. All data are shown as box and whiskers (*n* = 5 mice/group), student’s t test, *p < 0.05, **p < 0.01, ***p < 0.001.

Given the abundant expression of APP_swe_ in skeletal muscles, we wondered whether such muscular APP_swe_ expression could induce sarcopenia-like deficits, such as decreased muscle fiber size and increased muscle fiber degeneration [28, 29]. H&E histologic staining analysis of TA muscle fibers showed normal or comparable morphology in 3-MO *TgAPP*_*swe*_^*HSA*^ mice to those of control mice (Fig. 1A, B). However, at 6-MO, the mutant TA muscles exhibit sarcopenia-like deficits, showing smaller muscle fiber area with increased fibers containing central nuclei (Fig. 1A, B), a feature of muscle fiber degeneration [30]. We then characterized the phenotypes in other type of muscles, including quadricep and soleus. While the mutant quadricep, a proximal fast twitch type of muscles, showed similar age-dependent deficits to those of mutant TA muscles (Fig. 1C, D), the mutant soleus, a slow twitch type of muscles, exhibited an earlier onset degenerative phenotype, exhibiting a reduction in their fiber size, and an increase in fibers with central nuclei distribution at age of 3-MO (Fig. 1E, F), which were un-detectable in the mutant TA nor quadriceps at this age (Fig. 1). These results thus suggest muscle fiber type- and age-dependent sarcopenia-like deficits in *TgAPP*_*swe*_^*HSA*^ mice. This view was further tested by additional histologic staining analyses, including Gomori-trichrome, PAS (Periodic Acid Schiff), NADH-Transferase, and COX (cytochrome c oxidase), and SDH (succinate dehydrogenase), in both 3- and 6-MO TA and soleus muscles. Indeed, at both ages of 3- and 6-MO, the mutant soleus muscles showed increases in fibers with Gomori-trichrome positive staining, decreased COX^+^, but increased COX^-^:SDH^+^ fibers, and elevated cytoplasmic PAS^+^ fibers (Fig. S2), suggesting mitochondrial myopathy, fibrosis, and glycogen overload in the mutant soleus. The mutant TA muscles also showed decreased COX^+^ and increased COX^-^:SDH^+^ fibers at both 3- and 6-MO, elevated cytoplasmic PAS^+^ fibers at 6-MO, but little changes by Gomori-trichrome and NADH-TR staining (Fig. S2), suggesting a relatively weaker myopathy in TA than those in soleus muscles from the mutant mice. Together, these results provide additional support for earlier onset of myopathies in the mutant muscles, which resemble the features of sarcopenia-like myopathy.

### Age-dependent increases in hippocampal reactive astrocytes and microglia in *TgAPP*_*swe*_^*HSA*^ mice

We then asked whether *TgAPP*_*swe*_^*HSA*^ mice exhibit any brain pathology similar to those of APP_swe_-based AD animal models (e.g., *Tg2576*) [31–33]. It is known that APP_swe_-based AD animal models (e.g., *Tg2576*) exhibit not only increased Aβ_40_ and Aβ_42_ levels in the brain, but also elevated glial activation, inflammation, and reduced neuronal synapses [31–33]. We thus first measured both Aβ_40_ and Aβ_42_ levels in muscles, serum samples, and brain tissues in *TgAPP*_*swe*_^*HSA*^ mice (at 6-MO), compared with those of same aged *LSL-APP*_*swe*_ (a negative control) and *Tg2576* (a positive control) mice. ELISA analyses of Aβ_40_ or Aβ_42_ levels showed little to no Aβ increase in the hippocampus, cortex, or serum samples, but a slight increase in the TA muscles, of *TgAPP*_*swe*_^*HSA*^ mice (6-MO), as compared with those of the negative control mice (Fig. S1F, G). In contrast, Tg2576 mice showed marked increases of Aβ_40_ and Aβ_42_ levels in their brain tissues and serum samples, and a comparable level of Aβ_40_ in TA muscles to that of *TgAPP*_*swe*_^*HSA*^ mice (Fig. S1F, G).

We second examined neuronal distribution patterns and densities in the hippocampus and cortex of *TgAPP*_*swe*_^*HSA*^ mice (at 6-MO) through a co-immunostaining analysis using antibodies against NeuN (a marker for all neurons) and Ctip2 (a marker for neurons in the Layers V-VI cortex and CA1-2 and DG hippocampus). Little to no changes in the NeuN^+^ and Ctip2^+^ neuron distribution patterns and densities were detected in *TgAPP*_*swe*_^*HSA*^ brains, as compared with those of controls (Fig. S3).

**Fig. 2:**
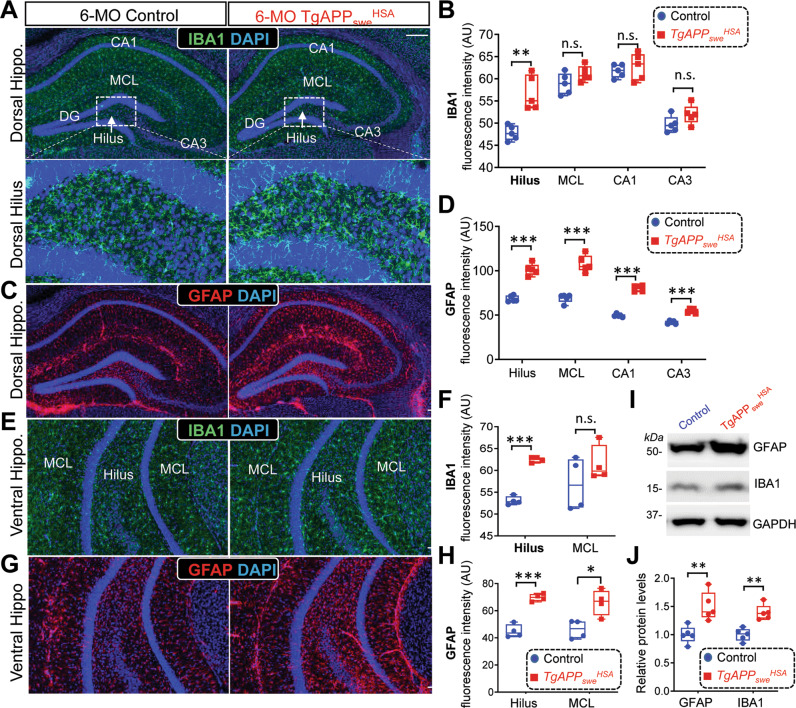
Elevated hippocampal reactivated astrocytes and microglial cells in 6-MO *TgAPP*_*swe*_^*HSA*^ mice. **A** Representative images of co-immunostaining with IBA1 (green) and DAPI (blue) of dorsal hippocampal sections from 6-MO control *(LSL-APP*_*swe*_) and *TgAPP*_*swe*_^*HSA*^ mice. **B** Quantification of the data in (**A**). **C** Representative images of co-immunostaining with GFAP (red) and DAPI (blue) of dorsal hippocampal sections from 6-MO control (*LSL-APP*_*swe*_) and *TgAPP*_*swe*_^*HSA*^ mice. **D** Quantification of the data in (**C**). **E** Representative images of co-immunostaining with IBA1 (green) and DAPI (blue) of ventral hippocampal sections from 6-MO control *(LSL-APP*_*swe*_) and *TgAPP*_*swe*_^*HSA*^ mice. **F** Quantification of the data in (**E**). **G** Representative images of co-immunostaining with GFAP (red) and DAPI (blue) of ventral hippocampal sections from 6-MO control (*LSL-APP*_*swe*_) and *TgAPP*_*swe*_^*HSA*^ mice. **H** Quantification of the data in (**G**). **I** Representative Western blots using antibodies against GFAP and IBA1 in homogenates of control and *TgAPP*_*swe*_^*HSA*^ hippocampus. GAPDH was used as a loading control. **J** Quantification of the data in (**I**). Scale bar: 100 μm. All quantifications of the data are presented as box and whiskers (*n* = 5). *p < 0.05, **p < 0.01, ***p < 0.001, student’s t test and correction of multiple comparisons using Holm-Sidak method was used, α set as 0.05.

Third, we assessed the morphologies and densities of glial cells, including Olig2^+^ oligodendrocytes, S100β^+^ ependymal cells, GFAP^+^ astrocytes, and IBA1^+^ microglial cells, in the brain sections of control (*LSL-APP*_*swe*_) and *TgAPP*_*swe*_^*HSA*^ mice. Again, little to no changes in the Olig2^+^ oligodendrocytes or S100β^+^ ependymal cells were detected in the brain of *TgAPP*_*swe*_^*HSA*^ mice (Fig. S4). However, both GFAP^+^ astrocytes and IBA1^+^ microglial cells were increased in the 6-MO *TgAPP*_*swe*_^*HSA*^ brain, particularly in the hippocampus at both dorsal and ventral regions (Fig. 2A–H), suggesting an activation of these glial cells. In line with this view, the increased GFAP and IBA1 protein levels were also detected in 6-MO *TgAPP*_*swe*_^*HSA*^ hippocampus using Western blot analysis (Fig. 2I, J). Notice that GFAP^+^ astrocytes and IBA1^+^ microglial cells were slightly increased in the cortex layer I-III of 6-MO *TgAPP*_*swe*_^*HSA*^ (Fig. S5A–C), but the protein levels remained unchanged in the 6-MO *TgAPP*_*swe*_^*HSA*^ cortex by Western blot analysis (Fig. S5D, E). This suggests that the hippocampus appeared to be more vulnerable than the cortex in the mutant mice. Negligible changes of these glial cells at 3-MO were observed in the mutant brain (Fig. S6), indicating an age-dependency of these phenotypes.

### Increases in expression of inflammatory cytokines, but decreases in growth factors and BBB integrity in the hippocampus of 6-MO *TgAPP*_*swe*_^*HSA*^ mice

Considering the tight association of glial cell activation with brain inflammation [13, 34], we examined expressions of inflammatory cytokines (e.g., *Il1b*, *Il6*, *Il10*, and *Tnfa*), chemokines (e.g., *Ccl3, 5, 12, 17*), and growth factors (e.g., *Pdgfb, Bdnf, Tgfb1* and *Csf2*) in the hippocampus of both control and *TgAPP*_*swe*_^*HSA*^ mice (at 3/6-MO) using RT-qPCR analysis (Fig. S7A, B). Among 12 genes examined in 3-MO *TgAPP*_*swe*_^*HSA*^ mice, only *Bdnf* was decreased, as compared with that of control mice (Fig. S7A). However, among the 61 genes examined in 6-MO *TgAPP*_*swe*_^*HSA*^ mice, 17 were increased and 8 were decreased in the mutant hippocampus (Fig. S7B). Interestingly, hippocampus of Tg2576 mice (6-MO) showed a similar inflammation phenotype to that of *TgAPP*_*swe*_^*HSA*^ mice, displaying increased expression of *Il6, Il15, Cxcl10, Lif*, and *Vegfd*, but decreased expression of *Pdgfb* and *Bdnf* (Fig. S7C, D).

**Fig. 3:**
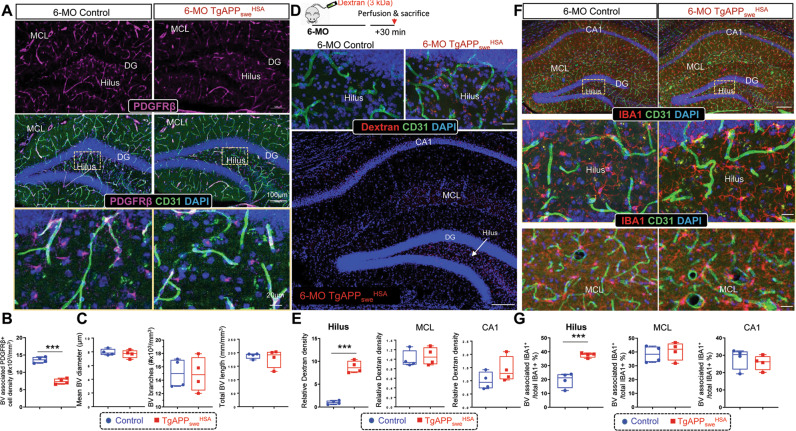
Decreased pericytes and increased leaky blood vessels and blood vessel associated microglial cells in the hippocampus of 6-MO *TgAPP*_*swe*_^*HSA*^ mice. **A** Representative images of co-immunostaining with CD31 (green), PDGFRβ (magenta), and DAPI (blue) of dorsal hippocampal sections from 6-MO control *(LSL-APP*_*swe*_) and *TgAPP*_*swe*_^*HSA*^ mice. Scale bars: 100 µm (upper) and 20 µm (lower). **B**, **C** Quantification of the data in (**A**). **D** Schematic diagram of the protocol for dextran injection and representative images of 3 kDa dextran (red) co-immunostained with anti-CD31 in 6-MO control *(LSL-APP*_*swe*_) and *TgAPP*_*swe*_^*HSA*^ hippocampi. Scale bars: 20 µm (upper) and 100 µm (lower). **E** Quantitative analysis of the data in (**D**). **F**, **G** Representative images of immunostaining using indicated antibodies (**F**) and the quantification (**G**) of blood vessel-associated microglia percentage in 6-MO control *(LSL-APP*_*swe*_) and *TgAPP*_*swe*_^*HSA*^ hippocampi. Scale bars: 100 µm (upper) and 20 µm (lower). Data are represented as box and whiskers (*n* = 4 mice/group). **p < 0.01, ***p < 0.001, student’s t test.

Notice that PDGF-BB is a key growth factor for the development of pericytes, a blood vessel associated cells that regulate BBB integrity [35, 36]. The reduction of *Pdgfb* in the mutant hippocampus led to a speculation for a deficit in PDGFRb^+^ pericytes. Indeed, co-immunostaining analysis showed decreased PDGFRβ^+^ pericytes, but little to no changes in the CD31 marked endothelial cells, in the mutant hippocampus (at 6-MO) (Fig. 3A–C). We then examined BBB leakage by tail vein injections of Dextran (3 kDa) into the control and mutant mice (at 6-MO) (Fig. 3D). The dextran signals outside of CD31^+^ blood vessels were detected in the mutant hippocampus, but not in the control (Fig. 3E). Interestingly, the dextran signals were largely in the mutant Hilus region (Fig. 3D), indicating a selective BBB leakage in this region. Moreover, more IBA1^+^ microglial cells were associated with CD31^+^ blood vessels in the mutant Hilus than those of the control mice (Fig. 3F, G), supporting the notion that blood vessel/BBB are damaged in this region.

### Age-dependent impairment in adult hippocampal neurogenesis in *TgAPP*_*swe*_^*HSA*^ mice

Given the reports that BDNF is a critical growth factor for adult hippocampal/ DG (dentate gyrus) neurogenesis [37–39], and considering the reduction of *Bdnf* in not only AD animal models [40], but also *TgAPP*_*swe*_^*HSA*^ hippocampus (Fig. S7), we examined DG neurogenesis in the mutant mice. EdU was injected into the mice (at ages of 1-, 3- and 6-MO, respectively, ~12 h before sacrifice) to label proliferative neural stem cells (NSCs). Hippocampal sections were co-immunostained with EdU and antibody against DCX (doublecortin), a marker for newborn neurons derived from NSCs, as shown in Fig. S8A. Remarkably, *TgAPP*_*swe*_^*HSA*^ mice at ages of 3-MO and 6-MO, but not 1-MO, displayed significant reductions in EdU^+^ and DCX^+^ cell densities at both dorsal and ventral DG (Fig. S8), demonstrating an early onset deficit in the NSC proliferation and thus DG neurogenesis in *TgAPP*_*swe*_^*HSA*^ mice, exhibiting another similar deficit as AD animal models [40].

### Age-dependent depression-like behaviors in *TgAPP*_*swe*_^*HSA*^ mice

**Fig. 4:**
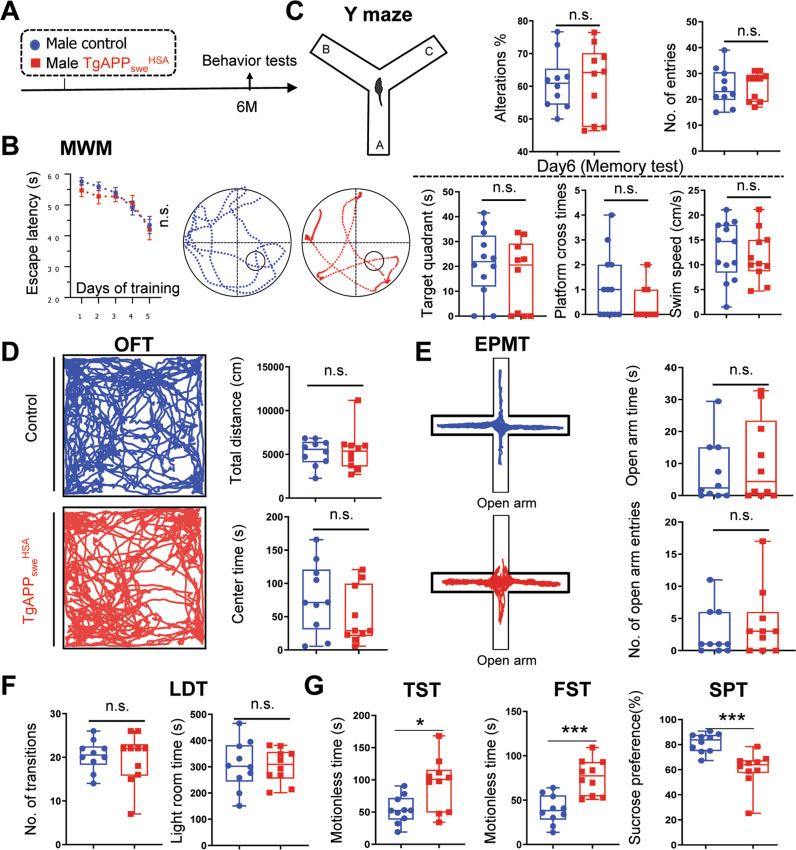
Depression-like behaviors in 6-MO *TgAPP*_*swe*_^*HSA*^ mice. **A** Schematic diagram of behavior tests. **B** MWM test: the latencies to reach the hidden platform during the training period, and the representative tracing images and quantification of time spent in target quadrant, platform crossing time, and swim speed in Day 6 memory test. **C** Y maze test: quantified alterations and number of total entries. **D** OFT: Representative tracing images and quantifications of the total distance traveled and the center duration time. **E** EPMT: Representative tracing images and quantifications of the open arm duration time and entries. **F** LDT: Quantification of the time spent in the light room and the number of transitions into the light room. **G** Quantified data of TST, FST, and SPT. In all these behavior tests, 6-MO control (*LSL-APP*_*swe*_) and *TgAPP*_*swe*_^*HSA*^ mice (males) were examined. All quantification data are shown as box and whiskers (*n* = 10 mice). *p < 0.05, **p < 0.01, ***p < 0.001, student’s t test and correction of multiple comparisons using Holm-Sidak method was applied in depression-related behaviors, α set as 0.05, adjusted *P* values were shown.

We then asked whether *TgAPP*_*swe*_^*HSA*^ mice show any behavior changes similar to those of AD animal models (e.g., *Tg2576*), such as age-dependent impairment in cognitive function [32, 33]. We first subjected *TgAPP*_*swe*_^*HSA*^ and control (*LSL-APP*_*swe*_) mice (at age of 6-MO, both males and females) to Morris water maze (MWM) and Y-maze, for the assessment of mouse spatial learning and memory function, and working memory, respectively [41, 42] (Figs. 4A–C and S9). No obvious difference in MWM or Y-maze task performance was observed between the mutant and control mice (Figs. 4A–C and S9), suggesting little cognitive decline in *TgAPP*_*swe*_^*HSA*^ mice.

Anxiety- and depression-like behaviors are often associated with increased glial activation and inflammatory cytokines, and decreased DG neurogenesis. We thus subjected mice (at ages of 3- and 6-MO) to behavior tests including open field test (OFT) to evaluate *TgAPP*_*swe*_^*HSA*^ mice’ locomotor activity, anxiety, and willingness to explore environments [43]; elevated plus maze test (EPMT) and light/dark transition test (LDT) to assess mouse anxiety-related behavior [43–45]; sucrose preference test (SPT), force swimming test (FST), and tail suspension test (TST) to examine mouse depression [45, 46]. As shown in Fig. 4D–G, *TgAPP*_*swe*_^*HSA*^ mice developed age-dependent depression-like behaviors. At age of 3-MO, no obvious difference in all the behavior tests was detected between mutant and control mice (Fig. S10). However, at 6-MO, the mutant male mice exhibited increased immobility times in both FST and TST, and decreased sucrose preference (Fig. 4G), without obvious changes in the performance of OPT, EPMT, and LDT (Fig. 4D–F), suggesting depression-like behavior, but little deficits in exploratory and locomotor activities and anxiety in 6-MO *TgAPP*_*swe*_^*HSA*^ mice. These depression-like behaviors were detectable not only in male, but also in female mutant mice (Fig. S9).

### Chronic systemic inflammation in *TgAPP*_*swe*_^*HSA*^ mice

**Fig. 5:**
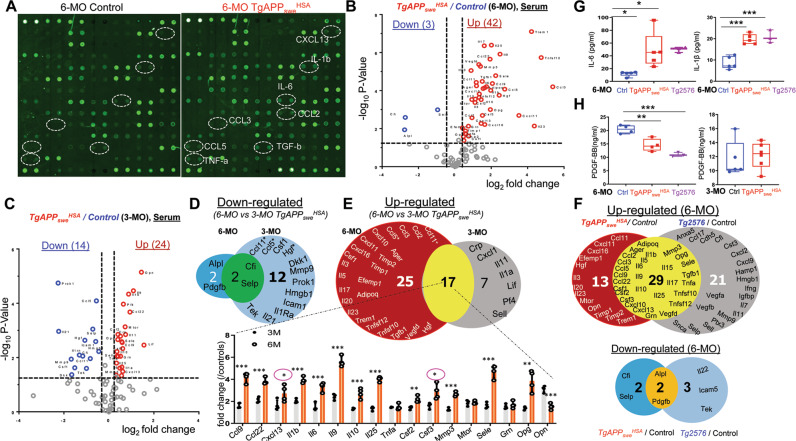
Increased systemic inflammation in *TgAPP*_*swe*_^*HSA*^ serum samples. **A** Representative images of serum L-Series label-multiplex antibody arrays of 6-MO control and *TgAPP*_*swe*_^*HSA*^ mice. **B** Volcano plots analysis of (**A**). **C** Volcano plots analysis of the serum L-Series label-multiplex antibody arrays of 3-MO control and *TgAPP*_*swe*_^*HSA*^ mice. **D**, **E** A comparison of the changes of downregulated (**D**) and upregulated (**E**) secreted factors in the antibody array between 3-MO and 6-MO. *p < 0.05, **p < 0.01, ***p < 0.001, student’s t test and correction of multiple comparisons using Holm-Sidak method was applied, circle with asterisk means no significant change was detected after correction. **F** A comparison of the changes of the up- and down-regulated secreted proteins in 6-MO Tg2576 over control mice with those detected in 6-MO *TgAPP*_*swe*_^*HSA*^ mice. **G** Elisa assays of serum IL1β and IL6 level in 6-MO control, *TgAPP*_*swe*_^*HSA*^ and *Tg2576* mice. The data are presented as mean ± SD (*n* = 5 mice). *p < 0.05, One-way ANOVA. **H** Elisa assays of serum PDGF-BB level in indicated mice. The data are presented as box and whiskers (*n* = 3–6 mice). *p < 0.05, One-way ANOVA and student’s t test were applied.

To understand how expression of APP_swe_ in skeletal muscles in TgAPP_swe_^HSA^ mice affects their brain inflammation and function, we tested a speculation that the inflammation and depression-like behavior phenotypes of *TgAPP*_*swe*_^*HSA*^ mice may be induced by secreted proteins from APP_swe_^+^ muscles. We first screened for altered serum plasma proteins in *TgAPP*_*swe*_^*HSA*^ mice (at 6-MO) using multiplex antibody-based arrays (Fig. 5A). Among the 90 proteins on the array, only 3 were lower, but 42 were higher in the mutant serum samples than those of control mice (Fig. 5A, B).

We second examined 3-MO mutant mice and compared the changes in their serum samples with those of 6-MO mutant mice. Using the same multiplex antibody-based arrays, 24 proteins were increased, and 14 were decreased in 3-MO mutant mice (Fig. 5C). Comparing the changes between 6-MO and 3-MO mutant samples, 17 proteins were increased, and 2 proteins were decreased at both 3- and 6-MO mutant serum samples (Fig. 5D, E). Interestingly, among these 17 increased proteins, 12 of them exhibited more dramatic increases in 6-MO than those of 3-MO mutant mice (Fig. 5E). These results suggest age-dependent changes in serum proteins in *TgAPP*_*swe*_^*HSA*^ mice.

Third, we addressed whether *TgAPP*_*swe*_^*HSA*^ mice (at 6-MO) exhibit similar changes in their serum samples to those of 6-MO Tg2576 mice. Remarkably, among 42 upregulated proteins in the serum of *TgAPP*_*swe*_^*HSA*^ mice, 29 were also elevated in *Tg2576* serum samples (Fig. 5F).

Finally, among the altered serum proteins in both *TgAPP*_*swe*_^*HSA*^ and Tg2576 mice, two pathways were noted: one is the increased pro-inflammatory cytokine (e.g., IL6 and IL1β) and chemokine pathway, and the other is the decreased growth factor (e.g., PDGF-BB) pathway. We thus used ELISA to further verified changes in IL6, IL1β, and PDGF-BB in serum samples of both mouse lines. Indeed, both IL6 and IL1β cytokines were increased in *TgAPP*_*swe*_^*HSA*^ and *Tg2576* serum samples (Fig. 5G); and PDGF-BB was significant lower in the serum samples of 6-MO *TgAPP*_*swe*_^*HSA*^ and *Tg2576* mice compared to those of control mice (Fig. 5H), but not in 3-MO *TgAPP*_*swe*_^*HSA*^ mice (Fig. 5H). Together, these results reveal a similar, but not identical, profile change in *TgAPP*_*swe*_^*HSA*^ serum samples to those of *Tg2576* mice, providing evidence for a chronic systemic inflammation in both *TgAPP*_*swe*_^*HSA*^ and *Tg2576*.

### Increased senescence and SASPs in TgAPP_swe_^HSA^ muscles

Given APP_swe_’s specific expression in skeletal muscles in *TgAPP*_*swe*_^*HSA*^ mice, we speculate that the APP_swe_^+^ muscles might be the key source for the increased systemic inflammation. We thus analyzed transcripts of the altered genes in the 6-MO control and *TgAPP*_*swe*_^*HSA*^ TA muscles. Among the 61 genes examined by RT-qPCR analyses, 37 up-, and 6 down-regulated transcripts were detected in *TgAPP*_*swe*_^*HSA*^ TA muscles (Fig. S11A). Interestingly, 49 factors were tested in both serum and TA muscle samples. 30 factors in serum and 27 factors in muscle were upregulated in mutant mice, and 19 (~63%) of them were increased in the mutant TA muscles (Fig. S11B). Comparing the altered transcripts between mutant muscles and hippocampus showed 12 upregulated transcripts (e.g., *Il6*, *Lif*, *Csf1*) and 3 downregulated transcripts (e.g., *Pdgfb*, *Bdnf,* and *Il4*) in both tissues (Fig. S11C, D). Whereas these results support the view for a systemic inflammation in the muscle-blood-hippocampus axis, further correlation plots showed a significant correlation of the significant changes of these transcripts between mutant TA muscles vs hippocampus (Fig. S11E), but not mutant TA muscles vs serum (Fig. S11F), nor mutant serum vs hippocampus (Fig. S11G).

**Fig. 6:**
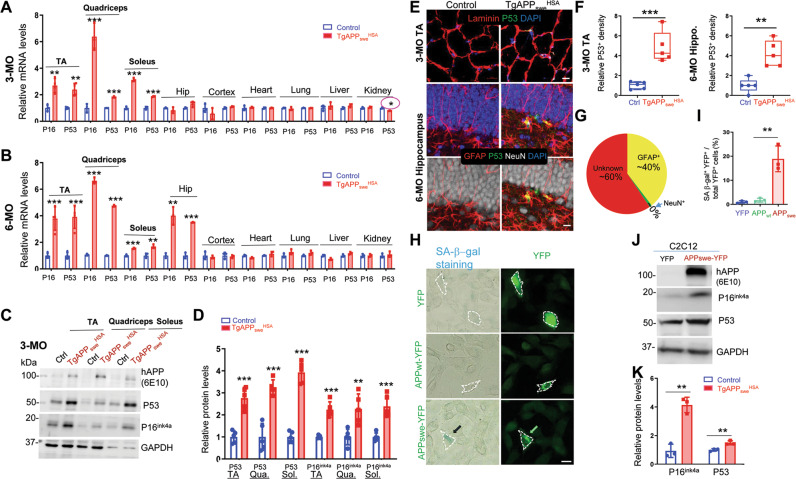
Increased cellular senescence in *TgAPP*_*swe*_^*HSA*^ skeletal muscles and APP_swe_^+^ C2C12 cells. **A**, **B** RT-PCR analysis of P16^ink4a^ and P53 gene expression in indicated tissues/organs of 3-MO (**A**) and 6-MO (**B**) control (*LSL-APP*_*swe*_) and *TgAPP*_*swe*_^*HSA*^ mice. **C**, **D** Western blot analysis of indicated protein expression in TA, quadriceps, and soleus from mice at 3-MO. GAPDH was used as a loading control. **C** Representative blots; and **D**, quantification analyses of the data in (**C**). **E** Representative images of co-immunostaining with p53 (green) with indicated antibodies in the 3-MO TA muscle and 6-MO hippocampus from control and *TgAPP*_*swe*_^*HSA*^ mice. Scale bars: 20 µm. **F**, **G** Quantification of the data in (**E**). **H** SA-β-gal staining of MC3T3 cells transfected with YFP, APP_swe_-YFP, APP_WT_-YFP, and YFP plasmids. Scale bar, 20 µm. **I** Quantification of SA-β-gal^+^ cell densities of data in **H** (*n* = 3 different cultures). **J** Representative Western blots using antibodies against indicated antibodies in homogenates of C2C12 cells transfected with YFP or APP_swe_-YFP. GAPDH was used as a loading control. **K** Quantification of the data in (**J**). Data in **F** are shown as box and whiskers. All the data are presented as mean ± SD, *n* = 3–5 mice/group, *p < 0.05, **p < 0.01, ***p < 0.001, by student’s t test in **A**, **B**, **D**, **F** and **K**, correction of multiple comparisons using Holm-Sidak method in **A**, **B** and **D**, and One-way ANOVA followed by Turkey’s multiple comparison post hoc test in (**I**). The circle with asterisk in (**A**)-means with significant difference, *P* < 0.05, by t-test, but no significant change after correction using Holm-Sidak.

Notice that the increased cytokines and chemokines in the mutant mouse muscle/serum/hippocampal samples exhibit features of senescence-associated secretory phenotype (SASP) [47, 48]. We thus asked if APP_swe_^+^ muscles exhibit increased expressions of senescence marker proteins, p16^Ink4a^ and p53. RT-qPCR analysis showed that both *p16*^*Ink4a*^ and *p53* were increased in all three muscles (TA, quadricepts, and soleus), but not in other tissues /organs of *TgAPP*_*swe*_^*HSA*^ mice at age of 3-MO (Fig. 6A). However, at age of 6-MO, in addition to these muscles, the mutant hippocampus, but not other tissues/organs, showed elevated expression of p16^Ink4a^ and p53 (Fig. 6B). These results suggest age-dependent and muscle and hippocampus selective cellular senescence. The increased muscle and hippocampal senescence in the mutant mice were further verified by Western blot analysis (Fig. 6C, D) and co-immunostaining analysis using antibodies against P53 (Fig. 6E–G and Fig. S12). Notice that the increased P53^+^ immunosignals were selectively detected in the mutant hippocampal hilus region, but not cortex of *TgAPP*_*swe*_^*HSA*^ mice (Fig. 6E–G and Fig. S12), in line with the view for the hilus region to be a more vulnerable region in response to the stress induced by the muscle APP_swe_. In addition, we verified the cellular senescence phenotype induced by expressing APP_swe_ in C2C12 cells (Fig. 6H–K). C2C12 cells (a muscle cell line) expressing APP_swe_-GFP, but not APP_wt_-GFP nor YFP showed increased SA-β-Gal (another marker for cellular senescence) (Fig. 6H. I), p16^Ink4a^, and p53 (Fig. 6J, K), indicating the specificity of the detrimental effect induced by the overexpression of APP_swe_.

### Attenuations of muscle and hippocampal pathologies, systemic inflammation, and behavior phenotypes in *TgAPP*_*swe*_^*HSA*^ mice treated with senolytic drugs

**Fig. 7:**
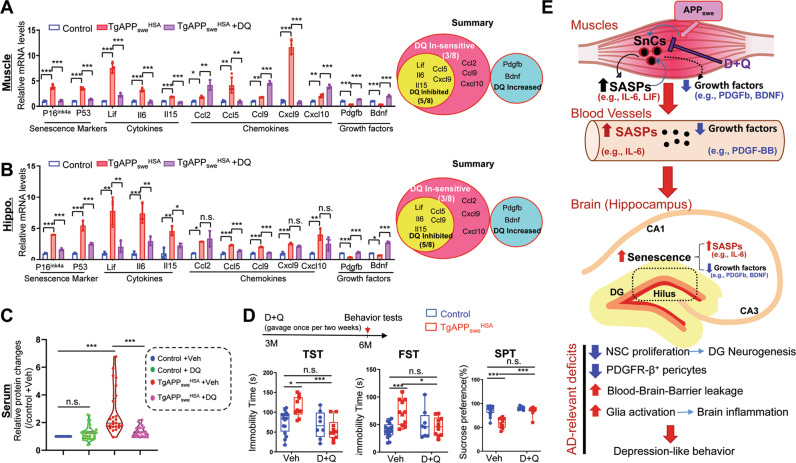
Attenuated depression-like behavior deficit in *TgAPP*_*swe*_^*HSA*^ mice treated with senescence inhibitors. **A**, **B** RT-PCR analysis of indicated gene expression in the TA muscles (**A**) and hippocampus (**B**) of 6-MO control and *TgAPP*_*swe*_^*HSA*^ mice with Veh or DQ treatments, data are present as mean ± SD, *p < 0.05, **p < 0.01, ***p < 0.001, *n* = 4 mice/group, one-way ANOVA followed by Sidak’s multiple comparisons post hoc test was used. **C** Summaries of DQ drugs’ effect on the factors tested in serum in Fig. S14. each dot represents different factors, and relative fold change over control mice treated with vehicle was shown, two-way ANOVA followed by Sidak’s multiple comparisons post hoc test was used. **D** A schematic diagram of the experimental design and the quantifications of depression-like behaviors using TST, FST, and SPT. 6-MO control (*LSL-APP*_*swe*,_, males) and *TgAPP*_*swe*_^*HSA*^ mice were treated with Veh (10%PEG 400) or DQ (D 5 mg/kg, Q 50 mg/kg, dissolved in 10% PEG 400, once per two weeks), starting at age of 3-MO, and then subjected to indicated behavior tests at 6-MO. All data are presented as mean ± SD. *p < 0.05, **p < 0.01, ***p < 0.001. *n* = 8–13 mice per group, two-way ANOVA followed by Sidak’s multiple comparisons post hoc test. **E** An illustration of the working model.

We next asked whether inhibition of senescence in *TgAPP*_*swe*_^*HSA*^ mice could diminish the brain and behavior phenotypes. A combination of Dasatinib (D) and Quercetin (Q) was used to inhibit senescence, due to their well examined senolytic effectiveness in animal studies [49, 50]. *TgAPP*_*swe*_^*HSA*^ mice at 3-MO were administered D + Q to then be subjected to phenotypic examinations at 6-MO (Fig. S13A). We first verified D + Q’s effect on muscle senescence in *TgAPP*_*swe*_^*HSA*^ mice. As shown in Fig. S13B, C, muscles from *TgAPP*_*swe*_^*HSA*^ mice treated with D + Q showed reduced expressions of senescence markers, p53 and p16^Ink4a^, confirming D + Q’s inhibitory effect. We also examined D + Q’s effect on various types of cells in TA muscles of *TgAPP*_*swe*_^*HSA*^ mice. In addition to muscle fibers, muscle tissue/organ contain nerve terminals (e.g., NMJ-neuromuscular junction), adipocytes, macrophages, and blood vessels [51, 52]. Oil Red O-stained adipocytes in the mutant TA muscles were comparable to that of controls (data not shown); however, CD11b^+^ macrophages were significantly increased in the mutant muscles (Fig. S13D, E). Interestingly, D + Q treatments abolished the increase of CD11b^+^ macrophages (Fig. S13D, E), increased muscle fiber size, and reduced central nuclei^+^ degenerative muscle fibers (Fig. S13F, G). Furthermore, we examined D + Q’s effect on SASP-like factors in muscles of *TgAPP*_*swe*_^*HSA*^ mice, which were largely reduced by the D + Q treatments (Fig. 7A). Together, these results suggest that muscle SnCs and the activated macrophages may contribute to the expression of these SASP factors as well as the sarcopenia-like muscle deficit.

We then determined whether the hippocampal phenotypes in *TgAPP*_*swe*_^*HSA*^ mice were diminished by D + Q treatments. Remarkably, the phenotypes including glial activation, elevated vessel associated microglia, increased SASP-like factors (e.g., *Lif*, *Il5*, *Il15*, *Ccl9*, and *Cxcl9*), and decrease of growth factors (e.g., *Pdgfd* and *Bdnf*) and PDGFRβ^+^ pericytes, in *TgAPP*_*swe*_^*HSA*^ hippocampus were all diminished by D + Q treatments (Fig. S14A–F and Fig. 7B). Moreover, the impaired hippocampal DG neurogenesis in *TgAPP*_*swe*_^*HSA*^ mice was restored by D + Q (Fig. S14G, H).

We further measured serum SASP-like cytokines and chemokines, and growth factors (e.g., PDGF-BB) in *TgAPP*_*swe*_^*HSA*^ mice with and without D + Q treatments. Notice that many cytokines (IL1b, IL3, IL4, IL7, IL23, TNFa, and TREM-1) and chemokines (CCL2, 3, 4, 4, 11, 12, 17, and CXCL10, 11, 13) were increased, and PDGF-BB was decreased in the serum samples of *TgAPP*_*swe*_^*HSA*^ mice treated with Vehicle (Fig. S15 and Fig. 7C). Those changes were all normalized by D + Q treatments (Fig. S15 and Fig. 7C), suggesting that the systemic inflammation in *TgAPP*_*swe*_^*HSA*^ mice is in large due to the APP_swe_-induced senescence and SASPs.

Finally, we compared behavior responses in *TgAPP*_*swe*_^*HSA*^ mice treated with or without D + Q. The depression-like behaviors by TST, FST, and SPT were also diminished in the mutant mice by D + Q treatments (Fig. 7D). Taken together, these results suggest that APP_swe_-induced senescence and SASPs are likely to prompt systemic and hippocampal inflammation, glial activation, and BBB leakage largely in the Hilus-hippocampus, which may underlie the depression-like behavioral phenotypes in 6-MO *TgAPP*_*swe*_^*HSA*^ mice (Fig. 7E and Table 1).

**Table 1:**
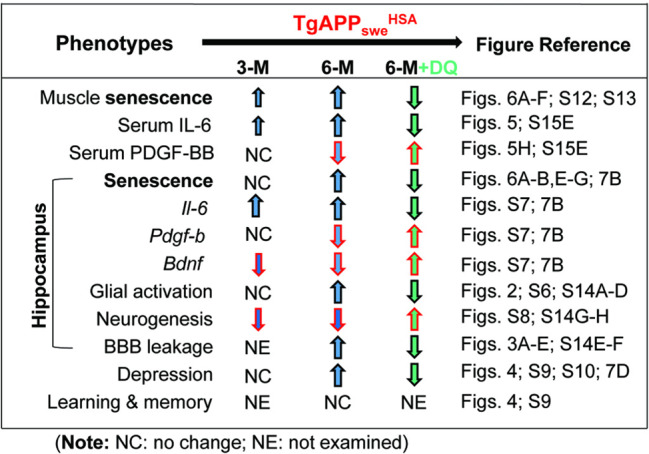
Summary of phenotypes in *TgAPP*_*swe*_^*HSA*^ mice .

## Discussion

Here, we use the *TgAPP*_*swe*_^*HSA*^ mouse model that selectively expresses APP_swe_ in skeletal muscles and provide evidence for muscular APP_swe_’s contributions to sarcopenia-like deficit, as well as AD-relevant brain pathology. We further investigated the mechanisms underlying muscular APP_swe_’s detrimental functions. Our results, summarized in Table 1, lead to a working hypothesis depicted in Fig. 7E, in which, muscular APP_swe_ promotes sarcopenia-like deficit, AD-relevant hippocampal pathology, and depression-like behavior likely due to the increased senescence and SASPs, which appear to be a driver for the systemic and hippocampal inflammation, and thus expedites hippocampus pathology and depression-like behaviors in *TgAPP*_*swe*_^*HSA*^ mice. These observations thus reveal a link of sarcopenia with AD, and uncover a muscular APP_swe_ to brain axis in AD development.

How does APP_swe_ in muscle cells induce brain/hippocampal pathology? We propose that APP_swe_-induced muscle senescence and SASPs may underlie its effects on the brain/hippocampus via systemic inflammation (Fig. 7E). Many SASP-like proteins were induced not only in APP_swe_^+^ muscles, but also in serum samples and hippocampus of *TgAPP*_*swe*_^*HSA*^ mice (Figs. 5, S7, and S11). Regarding the systemic inflammation in *TgAPP*_*swe*_^*HSA*^ mice, our results suggest that APP_swe_-induced senescence and SASPs in muscles appear to be a key contributor to this event. Many (19 over 30, ~63%) upregulated SASP-like factors are detected in both muscles and serum samples of TgAPP_swe_^HSA^ mice (Fig. S11B). Many (29 over 42, ~66%) increased serum proteins in TgAPP_swe_^HSA^ mice were also detectable in the Tg2576 mouse serum samples (Fig. 5F). Inhibition of senescence by its inhibitors (D + Q) abolished most of the increased inflammatory cytokines in the serum samples of TgAPP_swe_^HSA^ mice (Fig. S15 and Fig. 7C). However, further correlation analyses showed a significant association of SASP-like factors’ expression levels in *TgAPP*_*swe*_^*HSA*^ TA muscles with their hippocampus (Fig. S11E), but not with their serum samples (Fig. S11F). We speculate that such un-correlation may be due to the different muscle fibers with different phenotypes and different vulnerabilities to the APP_swe_ (Fig. 1 and Fig. S1C), which could express different SASP-like factors or cytokines, and thus make the elevated SASP-like factors more complex in *TgAPP*_*swe*_^*HSA*^ serum samples than those in *TgAPP*_*swe*_^*HSA*^ TA muscles. We also speculate that the dramatic effect on the systemic inflammation by APP_swe_ expression in muscles may be due to the abundant muscle tissues in the body, which account for 30–40% of a person’s body weight; and the consideration of muscles as a critical endocrine organ [53].

In addition, the hypothesis is also in line with our results that various types of muscles from *TgAPP*_*swe*_^*HSA*^ mice showed increased senescence cells as early as 3-MO (Fig. 6 and Fig. S12). Expression of APP_swe_, but not APP_wt,_ in C2C12 cells also increased senescence cells (Fig. 6H, I). These deficits occurred at the same age or earlier than the brain (largely hippocampus) phenotypes in *TgAPP*_*swe*_^*HSA*^ mice (Figs. 1, 2 and Fig. S8). Moreover, inhibition of senescence in *TgAPP*_*swe*_^*HSA*^ mice attenuated nearly all the hippocampal and behavior phenotypes (Fig. 7D and Fig. S14).

In addition to the increased SASP-like proteins (largely cytokines and chemokines), there are reductions in a few of growth factors, such as *Pdgfb* and *Bdnf* (Fig. S11A). The reduced *Pdgfb* and *Bdnf* were detected not only in muscles, but also in the hippocampus, of *TgAPP*_*swe*_^*HSA*^ mice (Fig. S7B). The decreased PDGF-BB was also observed in the serum samples of *TgAPP*_*swe*_^*HSA*^ mice (Fig. 5H). Interestingly, the inhibition of senescence restored PDGF-BB levels in *TgAPP*_*swe*_^*HSA*^ mice (Fig. S15E, F). These results suggest that APP_swe_ induced muscle senescence not only increases SASP-like proteins but also reduces these growth factors, which may also contribute to hippocampal pathology, especially BBB deficit, in the mutant mice.

In light of above observations, we speculate that the mutant muscle derived and increase pro-inflammatory cytokines (e.g., IL6) and decreased growth factors (e.g., BDNF and PDGF-BB) may play important roles in inducing cellular senescence, glial cell activation, and BBB deficit in the mutant hippocampus-in particular the Hillus region. Such a brain-region selective effect may be due to the abundantly expression of their receptors in hippocampal neurons, pericytes, and/or glial cells, which make hippocampus-Hillus region more vulnerable to the stress induced by these upregulated cytokines and/or downregulated growth factors. the receptors. In line with this view are reports that BDNF receptor-TrkB [54], PDGFRb [55], and cytokine receptor-check IL6’s receptor [56] are abundantly expressed in hippocampus. In addition, the hippocampal DG area has more neural stem cells, another feature making it more vulnerable to the stress-induced senescence. This view is also in line with multiple literature reports that link cellular senescence with muscle and brain aging, and various degenerative diseases, including AD [57–62]. Several papers also demonstrate the use of senolytic drugs to attenuate the disease progression in several AD animal models [63, 64]. However, how APP_swe_ in muscles induce senescence and SASPs remains unclear. We hope to address this question in future studies.

In summary, the results presented in this paper suggest a multi-cell and multi-organ model for AD development in which skeletal muscle cells may serve as a nidus of the disease. This study may reveal an important muscle-to-brain axis, where APP_swe_-induced muscle cell-senescence accelerates brain cell aging and neurodegeneration, opening new avenues to explore interactions between muscles and brain cells during AD development and progression.

## Supplementary information

Supplemental information

Supplemental table 2

Original western blots

checklist

## Data Availability

Data will be made available on reasonable request.
