# Supplementary material for: Muscular Swedish mutant APP-to-Brain axis in the development of Alzheimer’s disease
Source: Cell Death Dis. 2022 Nov 10;13(11):952. doi: 10.1038/s41419-022-05378-4 (PMC9649614; doi:10.1038/s41419-022-05378-4)
Supplement: Supplementary file 1 — Supplemental information [file 41419_2022_5378_MOESM1_ESM.docx]

**Supplemental information**

**This file contains other materials and methods and supplemental figures and figure legends.**

**Materials and methods**

**Animals**

The following mouse lines were used, *LSL-APP_swe_* and *TgAPP_swe_^HSA^* mice. In brief, the transcription of *hAPP_swe_* in *LSL-APP_swe_* mice is controlled by the CAG promoter, but its translation is blocked by a loxP-stop-loxP sequence (1). Thus, the expression of *hAPP_swe_* is controlled by both the *CAG* promoter and the *Cre*-dependent removal of *LSL*. The *HSA–Cre* mice were purchased from Jackson laboratory (donated by Dr. IMR Colony, stock #006149)(2). The *Tg2576* mice were purchased from Taconic, Hudson, NY, USA, which express human *APP695* with double mutations at *KM670/671NL (APP_swe_)* under the control of a hamster prion promoter (3). All mouse lines were backcrossed into C57BL/6 background. The C57BL/6J mice were bred in our laboratory using mice purchased from The Jackson Laboratory or obtained from the NIH aged rodent colony. All mice were housed in a room with a 12 h light/dark cycle and ad libitum access to water and rodent chow diet (P3000). The Case Western Reserve University Animal Resource Center monitors and maintains pathogen-free mouse housing. Control littermates were used in parallel for each experiment.

**Reagents**

The following primary antibodies were purchased from the following companies: Anti-hAPP (6E10, 803001, mouse) from Biolegend (San Diego, California, USA); Anti-DCX (SC-8066, goat) from Santa Cruz Biotech (Santa Cruz, California, USA); Anti-Ctip2 (ab18465, Rat), anti-Ki67 (ab16667, rabbit), anti-IBA1 (ab178846, rabbit and ab5076, goat), anti-P16^ink4a^ (ab211542, rabbit), and anti-P53 (ab26, mouse) from Abcam (Cambridge, Massachusetts, USA); Anti-S100β (287004, Guinea pig) from Synaptic System (Göttingen, Germany); Anti-Olig2 (p21954, rabbit) from Novus biologicals (Centennial, CO, USA); Anti-NEUN (12943S, rabbit), anti-APP (2452S, rabbit), anti-GFAP (12389S, rabbit), and anti-GAPDH (97166S, mouse) from Cell Signaling Technology (Danvers, Massachusetts, USA). Secondary antibodies were purchased from Jackson ImmunoResearch Laboratories (West Grove, Pennsylvania, USA). Dasatinib, quercetin, polyethylene glycol 400, DMSO, DAPI and 5-ethynyl-2’-deoxyuridine (EdU, a modified thymidine analogue that is incorporated into the DNA of dividing cells) were from Sigma Aldrich (St. Louis, MO, USA). All chemicals and reagents used in this study were of analytical grade.

The plasmids encoding YFP-APPswe mutation (K670N / M671L, AAG ATG - AAC TTG) was generated from the YFP-APP_WT_ construct utilizing the Q5 Site-Directed Mutagenesis Kit (E0554S, New England Biolabs, Inc) as described previously(4).

**Behavioral tests**

### Mice (male and female) at ages of 3- and 6-MO (month old) were used for behavioral studies.  Behavioral tests were carried out in blind to genotypes or treatments. For all behavioral experiments, mice were transferred to the testing room 2 hrs prior to the start of the test to allow mice to acclimate to the environment. All behavioral instruments were cleaned with 75% ethanol prior to each trial.

### The Morris water maze (MWM) was performed as previously described(5). Specifically, a 120 cm pool and a 10 cm platform were used for the water maze, and a nontoxic bright white gel (Soft Gel Paste Food Color, AmeriColor) was added to the water to make the surface opaque and to hide the escape platform (1 cm below the surface). Mice were trained for 5 days, four trials per day, with a 20 min interval between trials. For each trail, 60 sec was given for mice to locate the hidden platform. Eight spatial cues on the pool wall are visible for mice to find the hidden platform. On the 6^th^ day, the platform was removed, and the mice were placed into the pool at a new starting position. The time spent in each platform quadrant and the number of platform crossings within 60 sec were analyzed. The swim speed and the amount of time spent in each quadrant were quantified using the video tracking system (Noldus). The investigators were blind to genotype during data acquisition and analysis.

For Y- Maze test, each mouse was placed at the central of three opaque plastic arms and allowed to freely explore the three arms for 5 min. The total arm entries and spontaneous alternation were quantified.

### The open field test (OFT), elevated plus maze test (EPMT), and light/dark transition test (LDT) were performed as described previously(6). In brief, for OFT, each mouse was placed in a chamber (L x W x H = 50 x 50 x 20 cm) and its movement was monitored for 10 min using an overhead camera. Light intensity was about 150 lux. The video was analyzed by a tracking software (Etho Vision, Noldus). The total distance and the center (25 x 25 cm) duration time were quantified. For elevated plus maze test (EPMT), the EPM was placed 50 cm above the ground. Each mouse was initially placed in the center square facing one of the open arms (L x W = 60 x 5 cm). Light intensity was about 100 lux. Mice movement was recorded for 5 min using an overhead camera and tracking software (Etho Vision, Noldus). The amount of time spent in the open arms and the number of open arm entries were quantified. For light/dark transition test (LDT), each mouse was firstly placed in the dark compartment, overhead camera was turned on, and the door between lit and dark chambers was opened. The light intensity was about 200 lux in the lit chamber. 10 min of movement was recorded using a tracking software ((Etho Vision, Noldus). The time spent in the lit chamber and the number of transitions were quantified.

### The tail suspension test (TST), forced swimming test (FST), and sucrose preference test (SPT) were performed as described previously(7). For the TST and FST, the last 4-min of a 6-min test were analyzed, and the immobility time was measured directly. The sucrose preference test was carried out using a two-bottle choice procedure. Single housed mice were habituated to drink 2% (wt/vol) sucrose solution (dissolved in water) for 3 days, then mice were given access to the two pre-weighed bottles, one containing water and the other containing 2% sucrose solution. Bottle positions were changed every day, and water and sucrose solution consumption was assessed daily for 4 days. The consuming ratio of sucrose over total solution consumed was used for measuring the sucrose preference.

**Immunofluorescence staining and image analysis**

Immunostaining was performed as described previously(6). In brief, mice were anesthetized with isoflurane and transcardially perfused with PBS (50 mL) followed by 4% paraformaldehyde (PFA) in phosphate buffer (PBS) (pH 7.4) (50ml) to remove intravascular plasma proteins. The dissected brains were post-fixed in 4% PFA at 4 °C overnight. Coronal sections (50 μm) were washed with PBS (3 x 10 min) and treated with blocking reagent (5% Donkey Serum + 0.5% Triton 100×) for 1 h, then incubated overnight at 4 °C with the primary antibodies. On the second day, brain sections were washed 3 times and incubated with corresponding conjugated secondary antibodies and DAPI for 1 h. DAPI was used for nucleus counter staining. Stained sections were imaged using confocal microscope. Fluorescent quantification was performed using the ZEN software according to the manufacturer’s instructions (Carl Zeiss).

**Western blotting**

Western blotting was performed as described previously(8). Brain, muscle tissues, and cultured C2C12 cells were homogenized in RIPA buffer (50 mM Tris-HCl, pH 7.5, 150 mM NaCl, 1 mM EDTA,) containing 0.5% sodium deoxycholate, 0.1% SDS, 1 mM PMSF, 1 mM Na3VO4, 1 mM NaF, 1 mM DTT and protease inhibitor cocktail (Millipore, 539134). Then, the Lysates were centrifuged at 12,000 x g for 15 min at 4 °C to remove debris and to obtain homogenates. Samples were resolved by SDS-PAGE and transferred to a nitrocellulose membrane (1620112, Bio-Rad Laboratories). Then, the samples were incubated with 5% milk in TBST (10 mM Tris, 150 mM NaCl, and 0.5% Tween 20, pH 8.0) for 1 h. Next, membranes were immunoblotted with indicated antibodies at 4°C overnight. The membranes were washed with TBST three times and incubated with a 1:5,000 dilution of horseradish peroxidase–conjugated anti–mouse or anti–rabbit antibodies for 1 hour. Blots were washed with TBST 3 x 10 min and immunoreactive bands were visualized using the LI-COR Odyssey infrared imaging system. The intensity of immunoreactive bands were quantitated by using ImageJ (NIH).

**EdU injection and labeling**

### The control (*LSL-APPswe*) and *TgAPP_swe_^HSA^* mice (at 1-, 3 and 6-MO) were given four intraperitoneal injections of EdU (20 mg/kg/time, 1 time/4 h) within 12 h. 12 hours after their last injection, mice were euthanized and transcardially perfused with 50 ml of cold PBS and then with 50ml of 4% PFA. The dissected brains were post-fixed in 4% PFA at 4 °C overnight. In the next day, coronal sections (50 μm) were obtained for staining. EdU staining was performed using a Clik-iT EdU imaging kit with Alexa-Fluor 488 (Invitrogen) following the manufacturer's instructions.

**Stereological cell counting**

Stereological quantification of cells was performed as previously described(9, 10). Briefly, 40-μm thick brain sections were immunostained with related antibodies. One in every eight serial sections starting at the beginning of hippocampus (Bregma −1.06 mm) to the end of hippocampus (Bregma −3.80 mm) were examined with a Zeiss confocal system (LSM880), and each section was separated into ten z-plane images by a 4-μm step. About eight sections of each DG were counted, and total counts of eight examined sections were multiplied by 8 to estimate the total number of markers positive cells per DG. To quantify the cell density (number/volume area), we first measured the cell number of each section by using Image J software and calculated the volume as measured section area multiply thickness, then added up all cell number in each section, and then calculated the density as cell number per mm^3^.

**Histologic stains**

Indicated fast-twitch (e.g., TA and quadriceps) and slow-twitch (e.g., soleus) muscles from mice (male) at ages of 3- and 6-MO (month old) were isolated, and their frozen cross sections (10-12 μm) on cover slides were used for histological staining, including H & E, NADH-TR (nicotinamide adenine dinucleotide transferase), COX (Cytochrome c oxidase), SDH (succinate dehydrogenase), and Gomori-trichrome stains as described below.

The H & E staining was performed as described previously (11). In brief, the protocol is as below: a. Bring the slides to room temperature; b. Incubation of slides with Hematoxylin for 5 minute to stain the nuclei; c. Wash with 4-5 changes of tap water or until blue stops coming off slides; d. Counterstain in alcoholic-Eosin for 3-minute; e. Dehydrate through 2 changes of 95% EtOH for 20 sec each and 1 change of 100% EtOH 1 minute; f. Clear in 2 changes of Xylene 3 minute each change; and g. Mount with xylene-base mounting media and cover with coverslips.

For NADH-TR staining, muscle cryosections were rehydrated in PBS, then incubated in 0.4mg/ml of NADH and 0.8mg/ml nitroblue tetrazolium (NBT) in 0.5 M Tris buffer for 1hr at 37˚C. For SDH staining, the muscle sections were incubated in 100 mM sodium succinate salt and 1.2 mM NBT in 0.2 M phosphate buffer for 1 hr at 37˚C. The sections were then fixed, rinsed, washed with acetone solutions. For COX staining, the muscle sections were incubated with VitroView^TM^ COX Histochemistry Stain Kit (SKU# VB-3022) for 1hr at 37˚C. For modified Gomori-trichrome staining, the muscle sections were fixed at 4% PFA for 15 minutes and stained in Trichrome stain (Millipore Sigma, HT10) solution for 5 minutes then place in 0.5% acetic acid for 1 minute. For PAS staining, the muscle sections were fixed with Carnoy’s Fixative (60% of alcohol, 30% of chloroform, and 10% of glacial acetic). Samples were dehydrated and then stained with periodic acid and examined.

All stained muscle sections samples were mounted as described for H & E staining (e-g), and imaged at 20X magnification with BZX microscope.

**L-Series label-multiplex antibody arrays**

Blood samples were collected and allowed to clot for 30 min. They were then centrifuged for 10 min at 3000 rpm. The serum was aliquot and frozen at −80 °C until use. The antibody arrays were performed using an L-Series Glass Slide antibody arrays kit (AAM-SERV-LG, Raybiotech, USA) according to the manufacturer’s instructions as described previously(4, 12). In brief, the serum was dialyzed before biotin-labeling step. The primary amine of the proteins in the sample was biotinylated, followed by a dialysis to remove free biotin. The newly biotinylated sample was added onto the glass slide and incubated at room temperature. After incubation with Fluorescent Dye-Strepavidin, the signals were visualized by fluorescence.

**Mouse Cytokine Array**

Serum samples were collected as described above. Cytokines were measured with Mouse Cytokine Array Panel A (ARY006, R&D Systems) as described previously(8). In brief, the serum was mixed with a cocktail of biotinylated detection antibodies. The sample/antibody mixture was then incubated with the Mouse Cytokine Array membrane. Any cytokine/detection antibody complex present was bound by its cognate immobilized capture antibody on the membrane. Following three washes to remove unbound material, streptavidin–horseradish peroxidase and chemiluminescent detection reagents were added sequentially. The intensity of light was produced at each spot in proportion to the amount of cytokine bound.

**C2C12 cell culture and transfections of YFP, APP_WT_-YFP, APP_swe_-YFP plasmids**

### The C2C12 cells were grown in DMEM containing 20% (vol/vol) FBS, and 50 units/ml penicillin and streptomycin. Cells were transfected with control-YFP Vector, APP_WT_-YFP and APP_swe_-YFP by Lipofectamine 3000 (Invitrogen). 36 hours later, the transfected cells were transferred to 12-wells coverslips. 48 hours after transfection, the cells were subjected to SA-β-gal staining.

**SA-β -gal staining**

Cultured C2C12 cell SA-β-gal staining was performed as previously reported(8). The staining was performed using a SA-β-gal staining kit (Cell Signaling, #9860) according to the manufacturer’s instructions.

**Elisa assay for IL6, IL1β, PDGF-BB, human Aβ_40_ and human Aβ_42_**

Blood samples were collected, allowed to clot for 30 min, and centrifuged for 10 min at 3,000 rpm. The serum was aliquot and frozen at −80°C until use. Serum IL-6 was measured with the mouse IL-6 ELISA kit (550950, BD Biosciences) following the manufacturers’ instruction. Serum IL1β was measured with the Mouse IL-1 beta ELISA Kit (KE10003, Proteintech) following the manufacturers’ instruction. Serum PDGF-BB was measured with the Mouse/Rat PDGF-BB Immunoassay Kit (MBB00, R&D system) following the manufacturers’ instruction. Serum, brain, and muscle homogenization was obtained for human Aβ_40/42_ elisa assay. Brain tissues were homogenized as previously described(5). Human Aβ40 and Aβ42 level in serum, brain (300µg in total protein), and muscle (150µg in total protein) homogenates were measured using the Aβ_40_ human ELISA kit (Invitrogen, catalog #KHB3481) and the Aβ_42_ human ELISA kit (Millipore, catalog #EZHS42), respectively. Their concentrations were determined by comparing readings against the standard curves.

**RNA isolation and qPCR**

# Total RNA was isolated from brain tissues using the RNeasy Mini Kit (QIAGEN, Cat No. 74104). Total RNA of TA muscles was isolated by TRIzol extraction (15596018; Invitrogen). The purified RNA (1-5μg) was used for cDNA synthesis with GoScript™ Reverse Transcriptase Kit (Promega, # A5001). The cDNA products were subjected for subsequent quantitative PCR (qPCR) using a QuantiFast SYBR Green PCR Kit (204057; QIAGEN) with a qPCR System (StepOne Plus). Primers used are as listed in the Supplemental table 2. Each sample was repeated for at least 3 times, and the mRNA level was normalized to GAPDH using the 2-△△Ct method.

**Statistics and Reproducibility**

All data were expressed as mean ± SD Or box and whiskers. For in vivo studies, three to thirteen male mice per genotype per assay were used. For in vitro cell biological and biochemical studies, each experiment was repeated at least three times. Statistical analyses were performed using GraphPad Prism 7.0. The Mann-Whitney U test or unpaired student’s t test was used to compare data from two groups. For multiple comparisons of three or more groups of samples, the ANOVA test was used. For those have multi-parameter comparisons, correction for multiple comparison using Holm-Sidak method was applied, α was set as 0.05. The significance level was set at *P* < 0.05 (**P* < 0.05, ***P* < 0.01, ****P* < 0.001).

**References**

1. Xia WF, Jung JU, Shun C, Xiong S, Xiong L, Shi XM, et al. Swedish mutant APP suppresses osteoblast differentiation and causes osteoporotic deficit, which are ameliorated by N-acetyl-L-cysteine. J Bone Miner Res. 2013;28(10):2122-35.

2. Miniou P, Tiziano D, Frugier T, Roblot N, Le Meur M, Melki J. Gene targeting restricted to mouse striated muscle lineage. Nucleic acids research. 1999;27(19):e27.

3. Hsiao K, Chapman P, Nilsen S, Eckman C, Harigaya Y, Younkin S, et al. Correlative memory deficits, Abeta elevation, and amyloid plaques in transgenic mice. Science. 1996;274(5284):99-102.

4. Pan JX, Sun D, Lee D, Xiong L, Ren X, Guo HH, et al. Osteoblastic Swedish mutant APP expedites brain deficits by inducing endoplasmic reticulum stress-driven senescence. Commun Biol. 2021;4(1):1326.

5. Zhang H, Chen W, Tan Z, Zhang L, Dong Z, Cui W, et al. A Role of Low-Density Lipoprotein Receptor-Related Protein 4 (LRP4) in Astrocytic Abeta Clearance. The Journal of neuroscience : the official journal of the Society for Neuroscience. 2020;40(28):5347-61.

6. Sun D, Milibari L, Pan JX, Ren X, Yao LL, Zhao Y, et al. Critical Roles of Embryonic Born Dorsal Dentate Granule Neurons for Activity-Dependent Increases in BDNF, Adult Hippocampal Neurogenesis, and Antianxiety-like Behaviors. Biological psychiatry. 2020.

7. Sun D, Sun XD, Zhao L, Lee DH, Hu JX, Tang FL, et al. Neogenin, a regulator of adult hippocampal neurogenesis, prevents depressive-like behavior. Cell Death Dis. 2018;9(1):8.

8. Xiong L, Zhao K, Cao Y, Guo HH, Pan JX, Yang X, et al. Linking skeletal muscle aging with osteoporosis by lamin A/C deficiency. PLoS Biol. 2020;18(6):e3000731.

9. Sun XD, Chen WB, Sun D, Huang J, Li YQ, Pan JX, et al. Neogenin in Amygdala for Neuronal Activity and Information Processing. J Neurosci. 2018;38(44):9600-13.

10. Golub VM, Brewer J, Wu X, Kuruba R, Short J, Manchi M, et al. Neurostereology protocol for unbiased quantification of neuronal injury and neurodegeneration. Front Aging Neurosci. 2015;7:196.

11. Wang C, Yue F, Kuang S. Muscle Histology Characterization Using H&E Staining and Muscle Fiber Type Classification Using Immunofluorescence Staining. Bio Protoc. 2017;7(10).

12. Guo HH, Xiong L, Pan JX, Lee D, Liu K, Ren X, et al. Hepcidin contributes to Swedish mutant APP-induced osteoclastogenesis and trabecular bone loss. Bone Res. 2021;9(1):31.

13. Sugarman MC, Kitazawa M, Baker M, Caiozzo VJ, Querfurth HW, LaFerla FM. Pathogenic accumulation of APP in fast twitch muscle of IBM patients and a transgenic model. Neurobiology of aging. 2006;27(3):423-32.

14. Greenberg DS, Toiber D, Berson A, Soreq H. Acetylcholinesterase variants in Alzheimer's disease: from neuroprotection to programmed cell death. Neurodegener Dis. 2010;7(1-3):60-3.

15. Koistinen H, Prinjha R, Soden P, Harper A, Banner SJ, Pradat PF, et al. Elevated levels of amyloid precursor protein in muscle of patients with amyotrophic lateral sclerosis and a mouse model of the disease. Muscle & nerve. 2006;34(4):444-50.

16. Robertson TA, Dutton NS, Martins RN, Roses AD, Kakulas BA, Papadimitriou JM. Beta-amyloid protein-containing inclusions in skeletal muscle of apolipoprotein-E-deficient mice. Am J Pathol. 1997;150(2):417-27.

17. Zhang T, Shang R, Miao J. The role of amyloid beta in the pathological mechanism of GNE myopathy. Neurol Sci. 2022.

18. Ikezoe K, Furuya H, Arahata H, Nakagawa M, Tateishi T, Fujii N, et al. Amyloid-beta accumulation caused by chloroquine injections precedes ER stress and autophagosome formation in rat skeletal muscle. Acta Neuropathol. 2009;117(5):575-82.

19. Kuo YM, Kokjohn TA, Watson MD, Woods AS, Cotter RJ, Sue LI, et al. Elevated abeta42 in skeletal muscle of Alzheimer disease patients suggests peripheral alterations of AbetaPP metabolism. The American journal of pathology. 2000;156(3):797-805.

**Supplemental table 1: amyloid pathology in the (neuro)muscular diseases**

| **Muscle fiber type** | **Aβ/APP** | **Patients** | **Mouse models** |
| --- | --- | --- | --- |
| Type II fast-twitch fibers | Aβ accumulation | IBM(13) |  |
| Type IIB fast-twitch fibers | Aβ accumulation/increased hβAPP |  | IBM (MCK-βAPP) (14) |
| Deltoid muscles (fast-twitch) | increased APP | ALS(15) |  |
| Gastrocnemius (fast-twitch) | increased APP |  | ALS (G1H) (15) |
| TA (fast) and Soleus (slow-twitch) | Aβ accumulation |  | ApoE^-/-^ mice(16) |
| Skeletal muscles | Aβ accumulation | GNE(17) |  |
| Soleus (slow twitch) | Aβ accumulation |  | Chloroquine injection(18) |
| Temporalis | Aβ accumulation | AD(19) |  |

**Supplemental figures and figure legends**

**
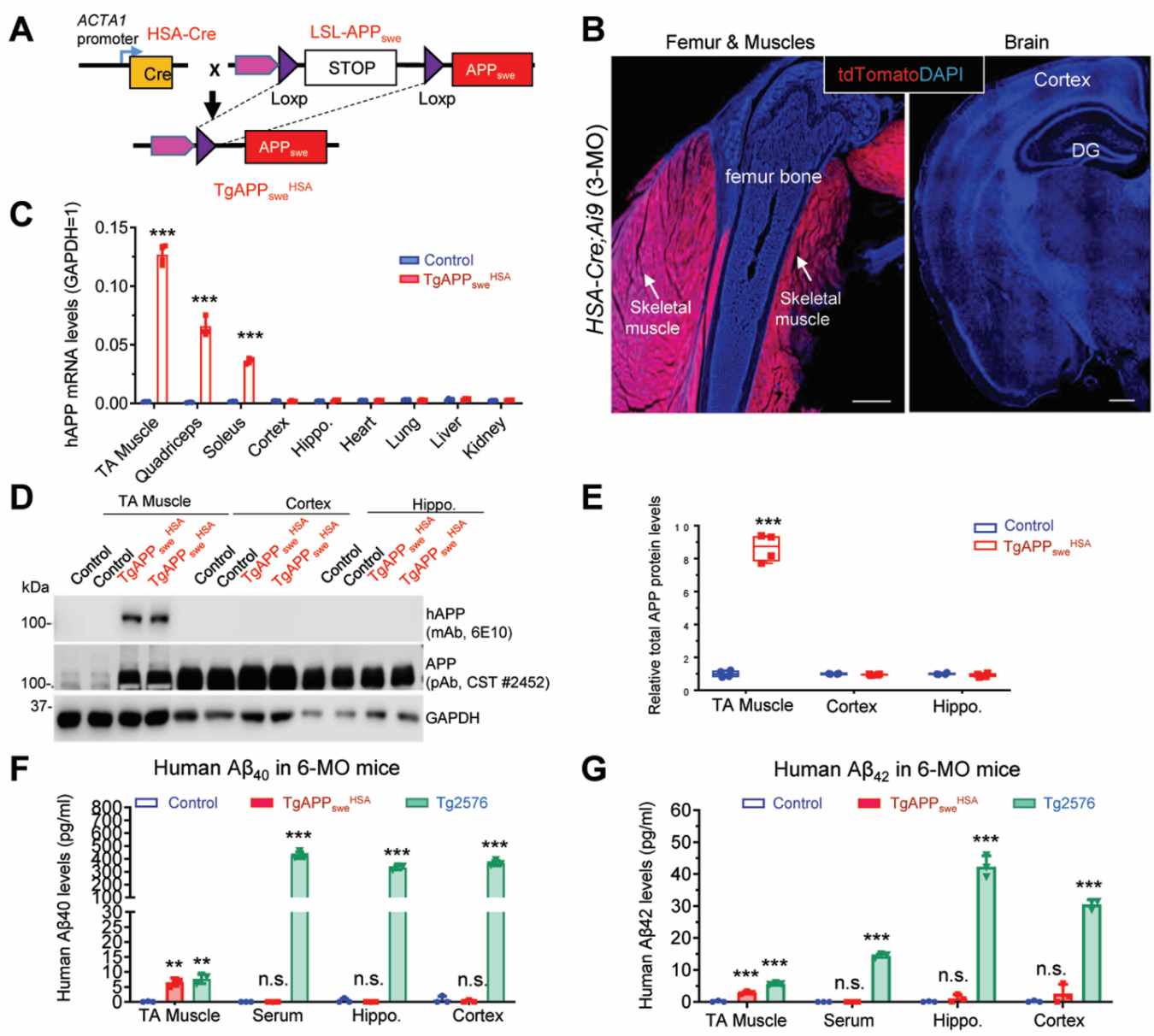
**

**Supplementary Fig. 1. Specific expression of APP_swe_ in skeletal muscles of *TgAPP_swe_^HSA^* mice. (A)** Illustration of the breeding protocol for the generation of TgAPP_swe_^HSA^ mice. **(B)** Representative fluorescence images of femur bone and muscle section from 3-MO *HSA-Cre; Ai9* mice*.* Tdtomato (Red) and DAPI (blue). Scale bar, 100*µ*m. **(C)** RT-PCR analysis of *hAPP* gene expression in the indicated tissues/organs of 6-MO control and *TgAPP_swe_^HSA^* mice. Data are present as mean ± SD, ***p < 0.001, n=4 mice in each group. **(D-E)** Western blot analysis of human APP (hAPP) and total APP protein levels in indicated tissues from 6-MO control (*LSL-APP_swe_*) and *TgAPP_swe_^HSA^* mice. **D**, representative blots; and **E,** quantification. **(F-G)** ELISA analyses of human Aβ_40_(**F**) and Aβ_42_(**G**) levels in serum, tibialis anterior (TA) muscles (150μg in total protein), and brain homogenates including hippocampus and cortex (300μg total protein) from 6-MO control, *TgAPP_swe_^HSA^*, and *Tg2576* mice. Data are presented as mean ± SD (n=3-4 mice). **p < 0.01, ***p<0.001 by Student’s t test for **C** and **E,** and by correction for multiple comparisons using the Holm-Sidak method and adjusted P value for **C**; and One-way ANOVA followed by Sidak multiple comparisons post hoc test for **F-G**.

**
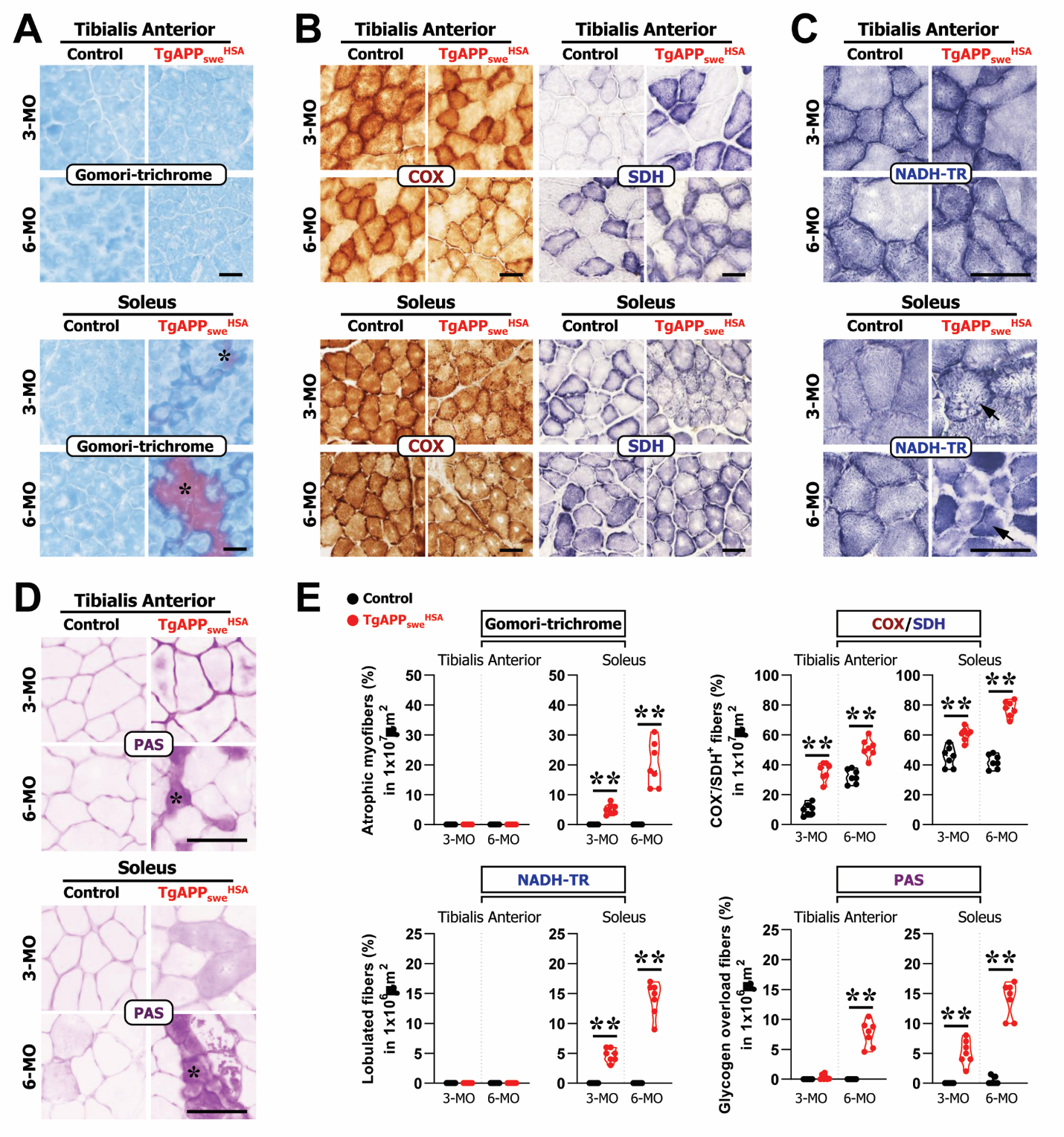
**

**Supplementary Fig. 2. Histological examinations of muscle fiber type- and age-dependent pathology in *TgAPP_swe_^HSA^* mice**. Representative images are TA (distal fast-twitch muscle) and soleus (slow-twitch muscle) from *TgAPP_swe_^HSA^* mice and littermate control mice at the indicated age. **(A)** Representative images of histological staining with modified Gomori trichrome showing atrophic myofibers filled with red staining in *TgAPP_swe_^HSA^* soleus muscles. The black star (*) indicates the atrophic myofiber. **(B)** Representative images of staining with COX (Cytochrome C Oxidase) and SDH (Succinate dehydrogenase), respectively. **(C)** Representative images of staining with NADH-TR (nicotinamide adenine dinucleotide transferase). The lobulated fiber (the black arrow) were showing in the soleus at the indicated age in *TgAPP_swe_^HSA^*. **(D)** Representative images of staining with PAS (Periodic acid Schiff). Glycogen overload, indicated by the black star (*), was detected in the TA and soleus at the indicated age in *TgAPP_swe_^HSA^*. Scale bar = 50 µm. **(E)** Quantification analyses. The average intensities were quantified and normalized to the intensity of the control. Data were analyzed by student’s t-test, Data are presented as mean ± SD (n=3 mice). **p<0.01, compared to control.


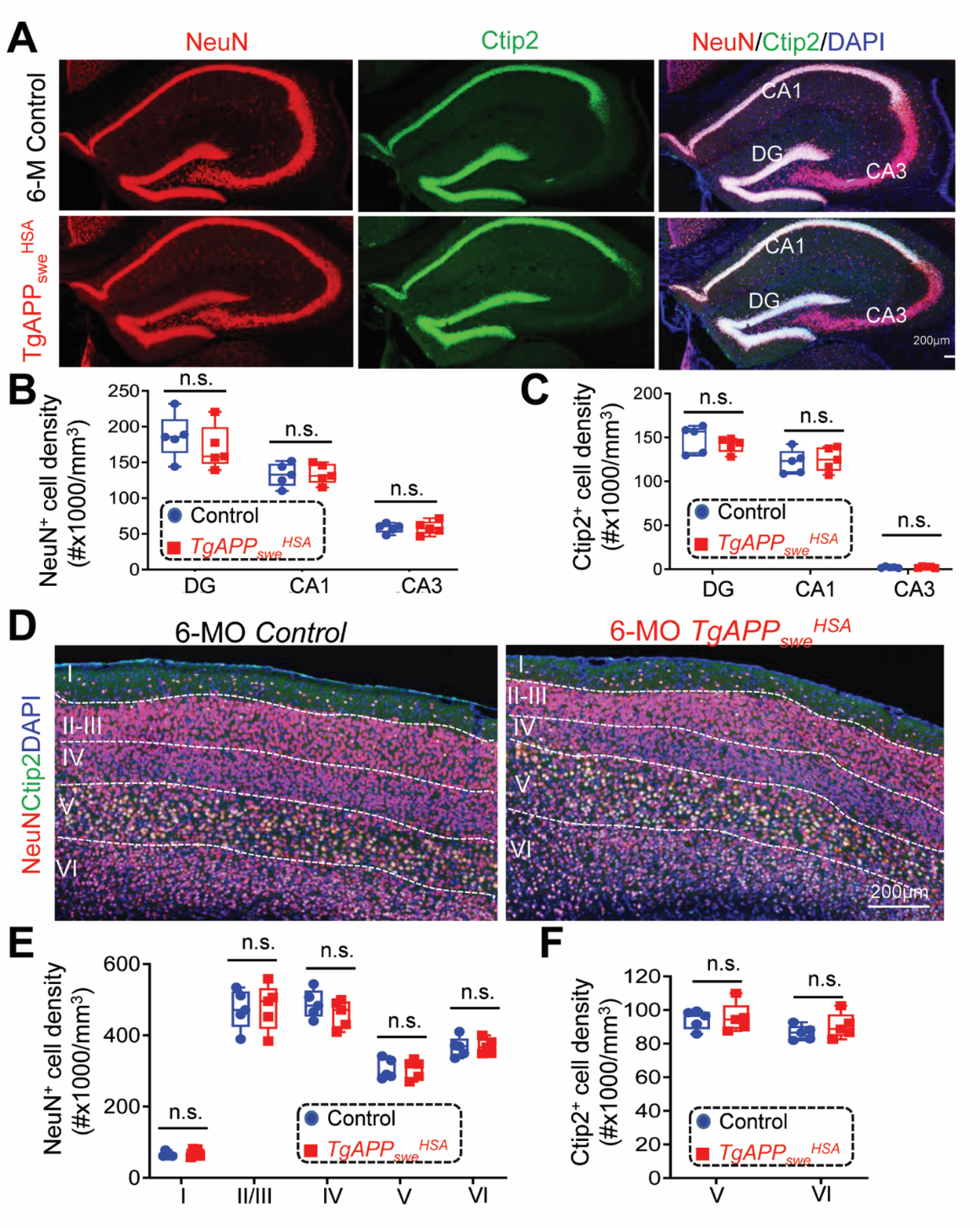


**Supplementary Fig. 3. Normal neuron distribution pattern and densities in the cortex and hippocampus of *TgAPP_swe_^HSA^* mice. (A)** Representative images of dorsal hippocampal sections from 6-MO control and *TgAPP_swe_^HSA^*, coimmunostained with Ctip2 (green), NeuN (red), and DAPI (blue). Scale bar, 200µm. **(B-C)** Quantification analyses of NeuN cell densities in GCL, CA1, CA3 in **A**. **(D)** Representative images of cortical sections from 6-MO control and *TgAPP_swe_^HSA^,* coimmunostained with Ctip2 (green), NeuN (red), and DAPI (blue). Scale bar, 200µm. **(E-F)** Quantification analyses of NeuN cell densities in different cortical layers (**E**) and Ctip2 cell densities in V and VI layer (**F**) shown in **D**, n=5. All data are presented as box and whiskers, no significant difference was detected by student’s t test.


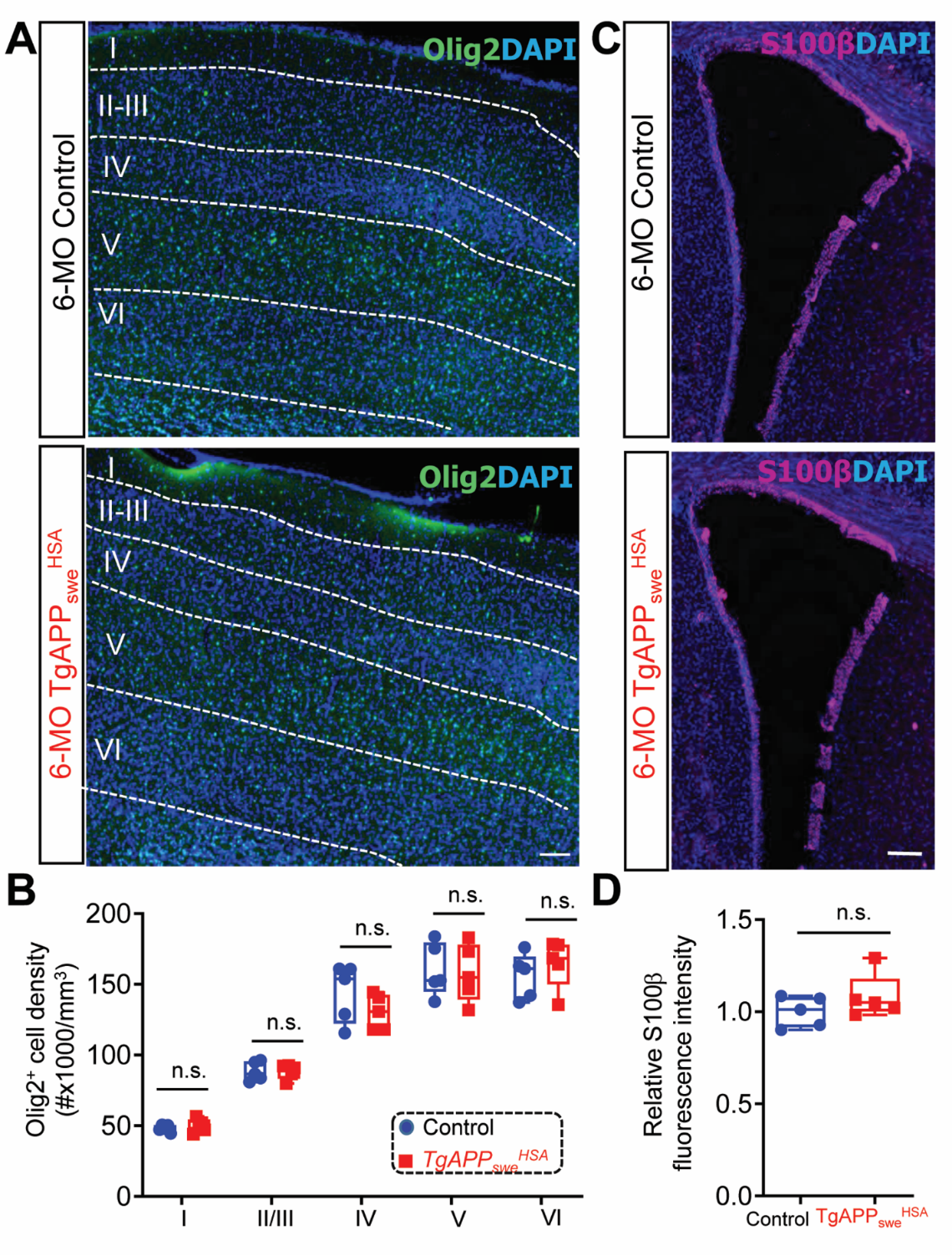


**Supplementary Fig. 4. Normal Olig2^+^ oligodrocytes and S100β^+^ ependymal cells in 6-MO *TgAPP_swe_^HSA^* mice. (A)** Representative images of cortical sections from 6-MO control and *TgAPP_swe_^HSA^* mice coimmunostained with Olig2 (green) and DAPI (blue). Scale bar, 100µm. **(B)** Quantification analyses of cortical Olig2^+^ cell densities in **A**, n=5. **(C)** Representative images of subventricular zone sections from 6-MO control and *TgAPP_swe_^HSA^* mice coimmunostained with S100β (magenta), and DAPI (blue). Scale bar, 100µm. **(D)** Quantification analyses of S100β^+^ fluorescence intensity in **C**. n=5. All data are presented as box and whiskers, no significant difference was detected, student’s t test.


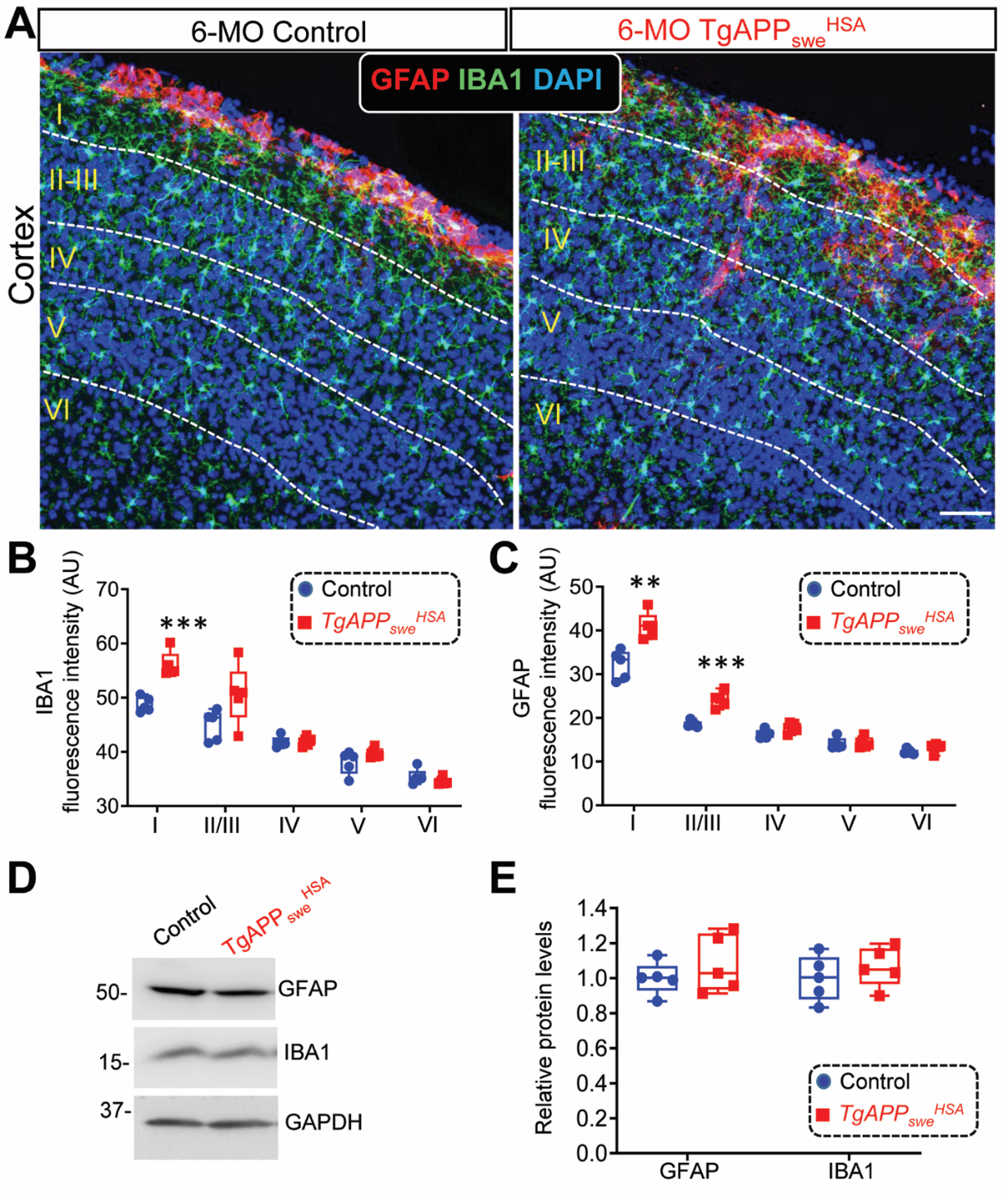


**Supplementary Fig. 5. Slightly elevated reactive astrocytes and microglial cells in 6-MO *TgAPP_swe_^HSA^* cortex layerI-III.**

**(A)** Representative images of co-immunostaining with IBA1 (green), GFAP (red), and DAPI (blue) of cortex sections from 6-MO control (*LSL-APP_swe_*) and *TgAPP_swe_^HSA^* mice. Scale bar: 100µm. **(B-C)** Quantification of the data in **A**. **(D)** Representative Western blots using antibodies against GFAP and IBA1 in homogenates of cortex of control and *TgAPP_swe_^HSA^* mice. GAPDH was used as a loading control. **(E)** Quantification of the data in **D**. All data are present as box and whiskers, n=5, **p<0.01, ***p<0.001 by student’s t test; in **B** and **C**, the correction of multiple comparisons using Holm-Sidak method was used, and the adjusted P values were shown.


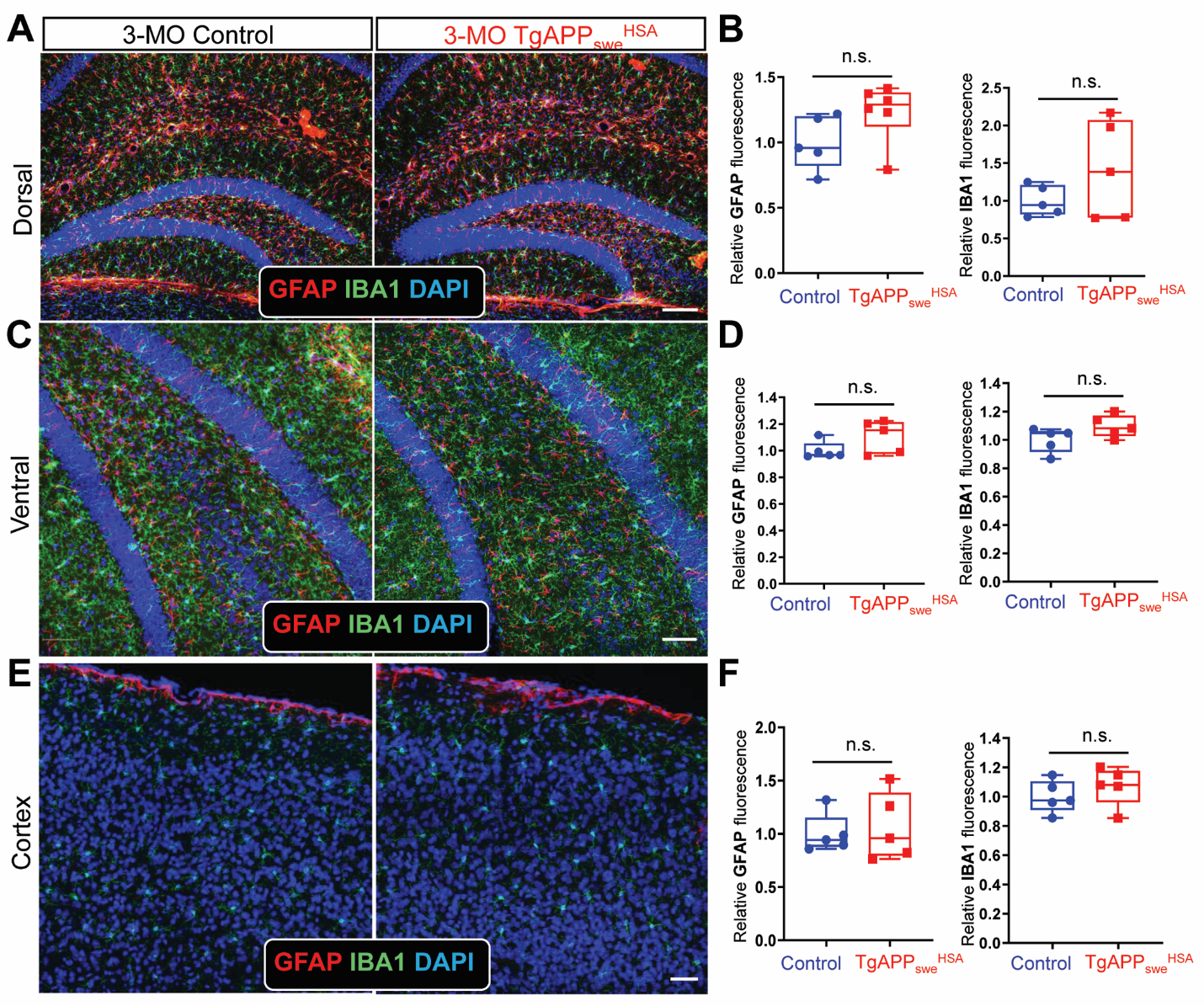


**Supplementary Fig. 6. Normal GFAP^+^ astrocytes and IBA1^+^ microglial cells in 3-MO *TgAPP_swe_^HSA^* mice.** **(A, C, E)** Representative images of dorsal**(A)**, ventral**(C)** hippocampal sections and cortical sections**(E)** from 3-MO control and *TgAPP_swe_^HSA^* mice coimmunostained with IBA1 (green), GFAP (red), and DAPI (blue). Scale bar, 100µm. **(B, D, F)** Quantification analyses of the dorsal**(B)**, ventral**(D)** hippocampal and cortical **(F)** GFAP and IBA1 fluorescence intensity data in **A, C, E** respectively. All the data are presented as box and whiskers, n=5, no significant difference was detected by student’s t test.


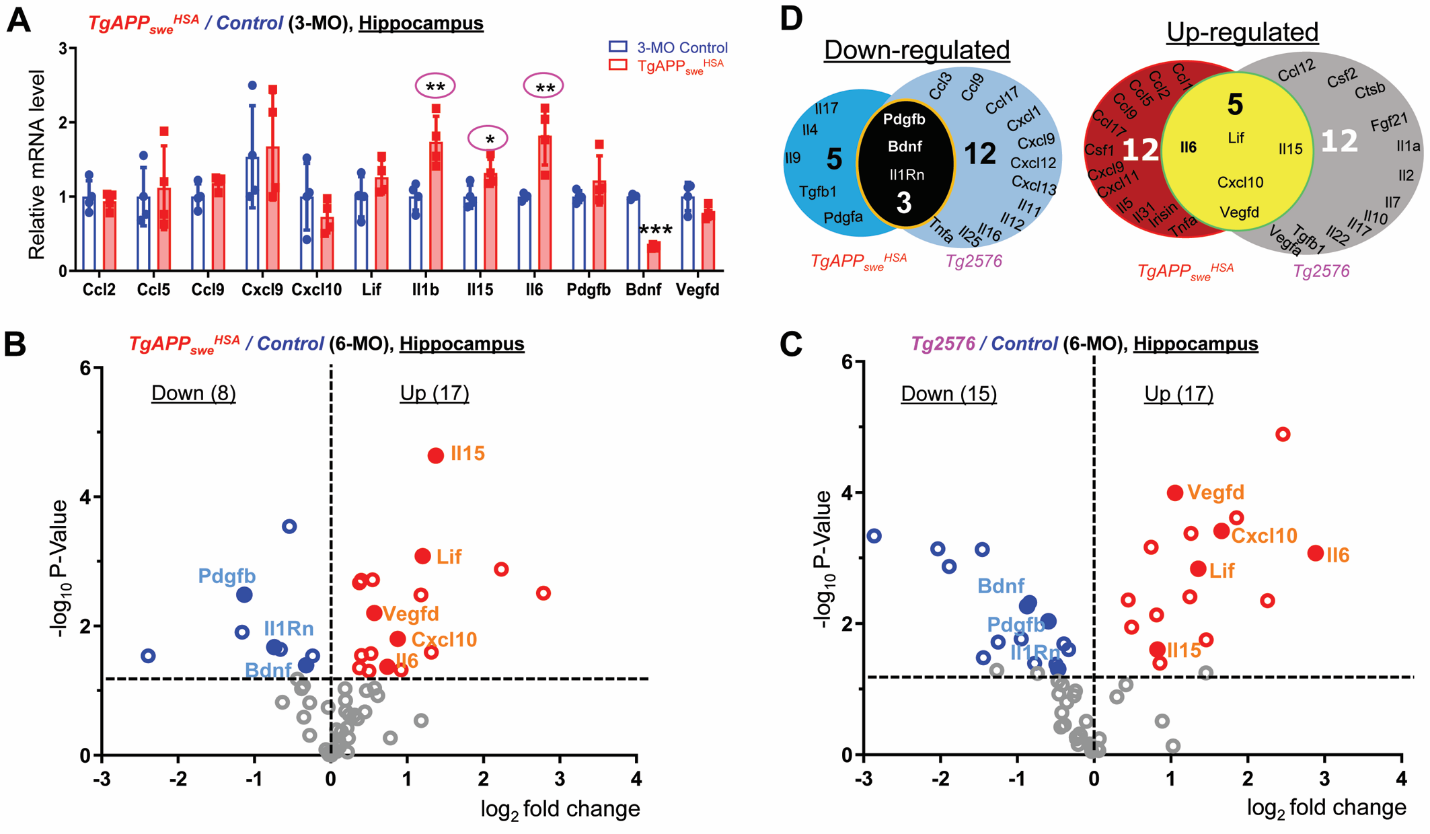


**Supplementary Fig. 7. Increased inflammatory factors in the hippocampus of 6-MO *TgAPP_swe_^HSA^* mice. (A)** RT-PCR analysis of indicated gene expressions in the hippocampus of 3-MO control and *TgAPP_swe_^HSA^* mice, data are present as mean ± SD, n=4 mice in each group, *p < 0.05, **p < 0.01, ***p < 0.001, by student’s t test, and correction for multiple comparisons using Holm-Sidak method was also used. Note, circle with asterisk indicating no significant difference after correction. **(B-C)** Volcano plots analysis of changes in mRNA levels of 61 genes in 6-MO *TgAPP_swe_^HSA^* **(B)** and Tg2576 **(C)** hippocampi. *p<0.05 **(D)** Comparison analysis of mRNA expressions (up-regulated and down-regulated) between *TgAPP_swe_^HSA^* and Tg2576 hippocampi**.**

**
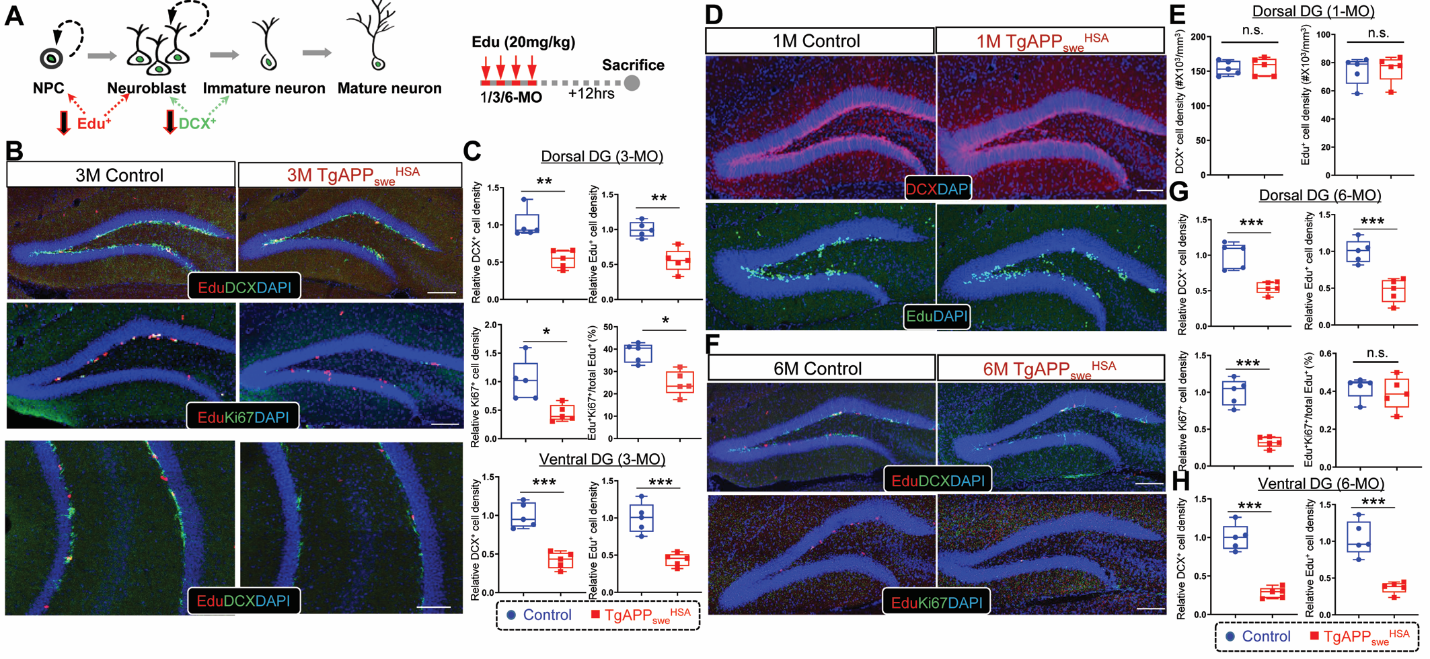
Supplementary Fig. 8. Reduced hippocampal neurogenesis in 3- and 6-MO but not 1-MO *TgAPP_swe_^HSA^* mice. (A)** Schematic diagram of the protocol for Edu injections. **(B)** Representative images of double immunostaining of Edu (red) and DCX (green) or Ki67(green) in dorsal and ventral DG from 3-MO control and *TgAPP_swe_^HSA^* mice. Scale bar, 100*µ*m. **(C)** Quantification of the data in **B**. **(D)** Representative images of double immunostaining of Edu (green) or DCX (red) with DAPI in dorsal DG from 1-MO control and *TgAPP_swe_^HSA^* mice. Scale bar, 100*µ*m. **(E)** Quantification of the data in **D**. **(F)** Representative images of double immunostaining with Edu (red) and DCX (green) or Ki67(green) in the dorsal DG of 6-MO control and *TgAPP_swe_^HSA^* mice. Scale bar, 100*µ*m. **(G)** Quantification of the data in **F**. **(H)** Quantification of DCX+ and Edu+ cell density in the ventral DG of 6-MO control and *TgAPP_swe_^HSA^* mice. All quantification data are presented as box and whiskers (n=5 mice/group). *p<0.05, **p<0.01, ***p<0.001 by student’s t test.


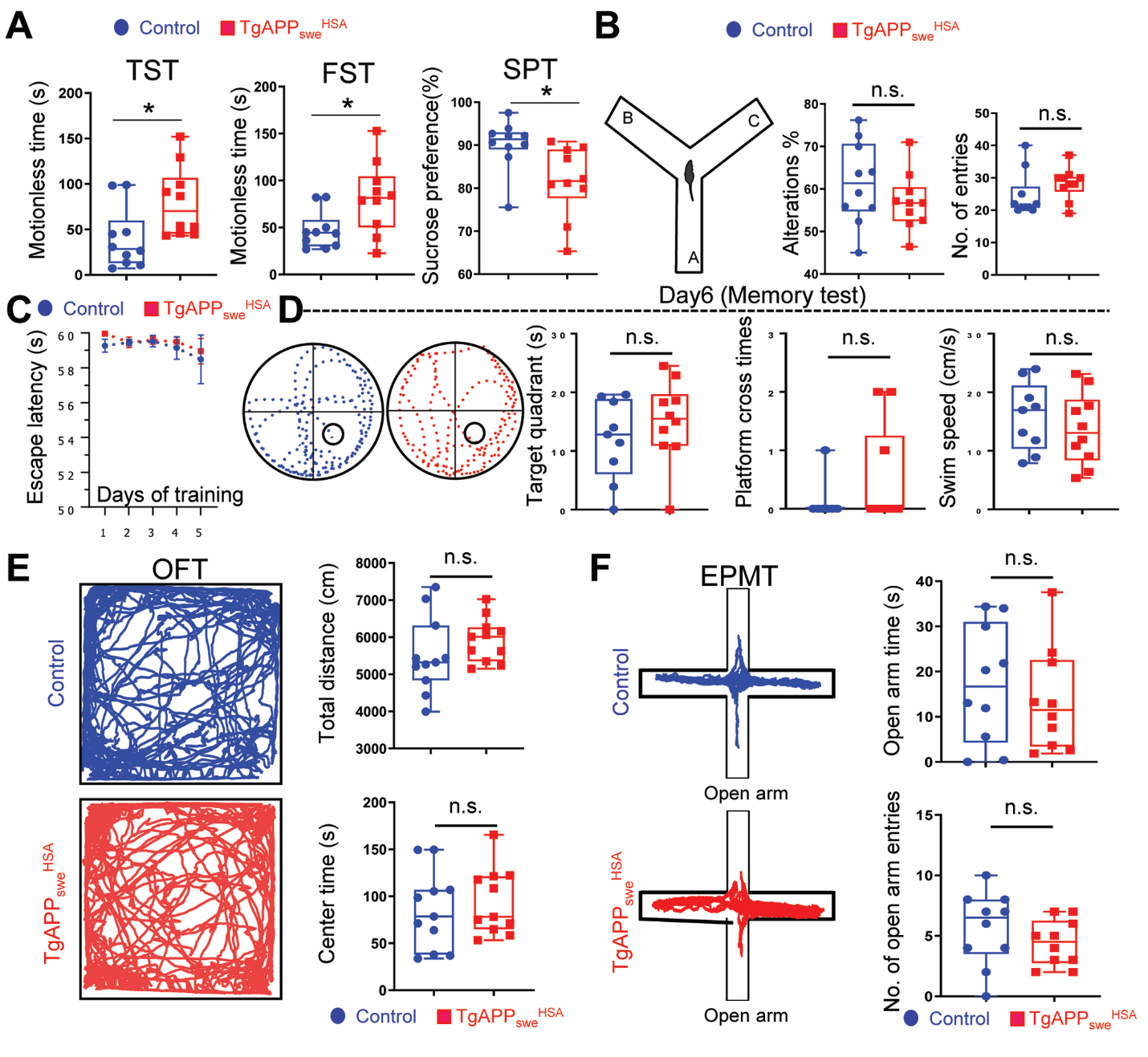


**Supplementary Fig. 9. 6-MO female *TgAPP_swe_^HSA^* mice showed depression-like behaviors in *TgAPP_swe_^HSA^* mice. (A)** Quantified data of TST, FST, and SPT, student’s t test and correction for these three behavior comparisons using Holm-Sidak method were performed, adjusted P values were shown. **(B)** Y maze test: quantified alterations and number of total entries. (**C-D**) MWM test: the latencies to reach the hidden platform during the training period, (**C**)**,** and the representative tracing images and quantification of time spent in target quadrant, platform crossing time, and swim speed(**D**). **(E)** OFT: Representative tracing images and quantification of total distance and center duration time. **(F)** EPMT: Representative tracing images and quantifications of open arm duration time and entries**.**  In all these behavior tests, 6-MO control (*LSL-APP_swe_*) and *TgAPP_swe_^HSA^* mice (females) were examined. All quantification data are shown as box and whiskers (n=10 mice), *p<0.05, student’s t test.


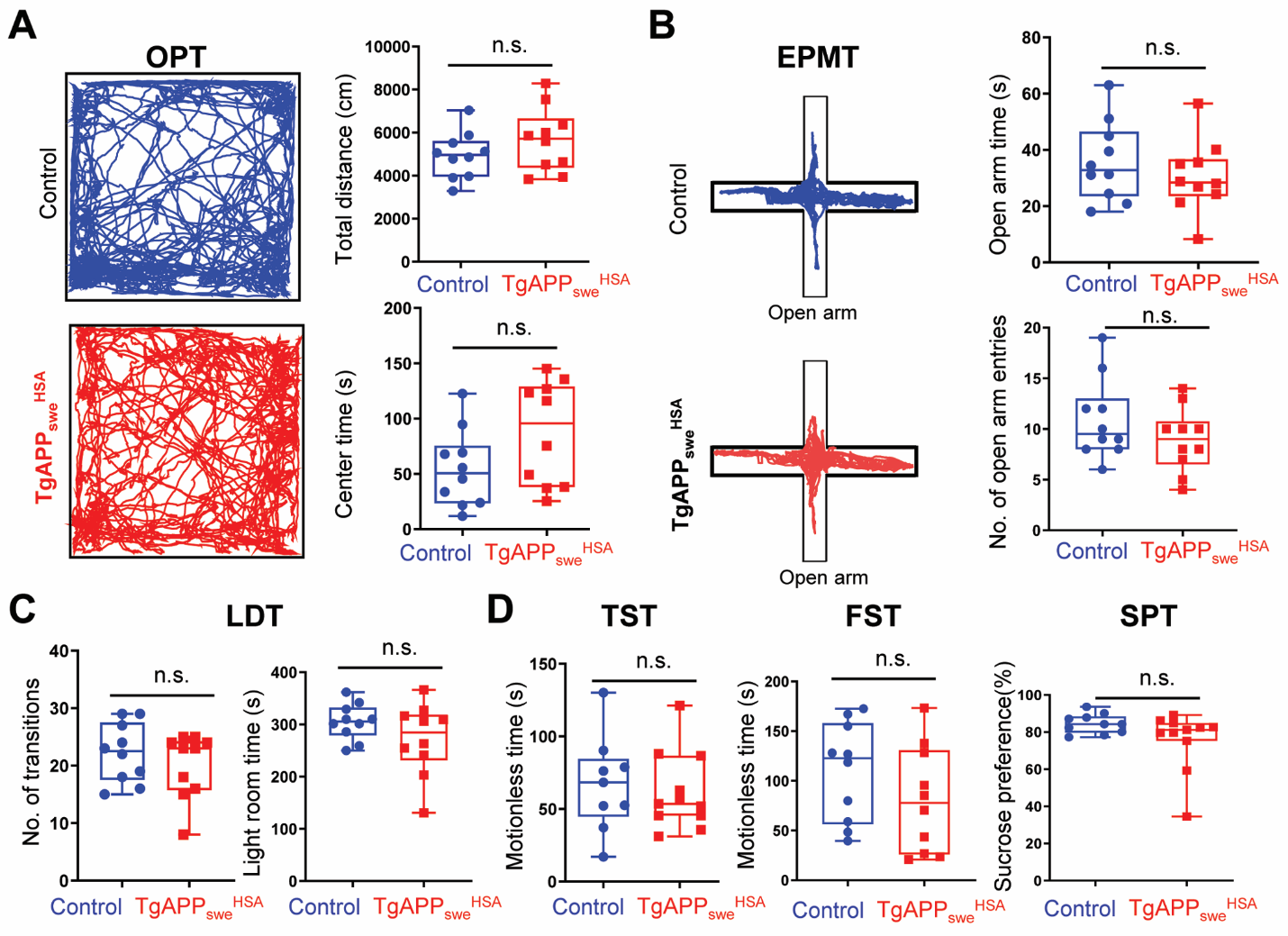


**Supplementary Fig. 10. Normal depression-like and anxiety-like behaviors in 3-MO *TgAPP_swe_^HSA^* mice. (A)** OFT: Representative tracing images and quantifications of total distance and center duration time. **(B)** EPMT: Representative tracing images, and quantifications of open arm duration time and entries**. (C)** LDT: Quantification of the time spent in the light room and the number of transitions into the light room. **(D)** Quantified data of TST, FST, and SPT. In all these behavior tests, 3-MO control (*LSL-APP_swe_*) and *TgAPP_swe_^HSA^* mice (males) were examined. All quantification data are shown as box and whiskers (n=10 mice), no significance was detected by student’s t test.


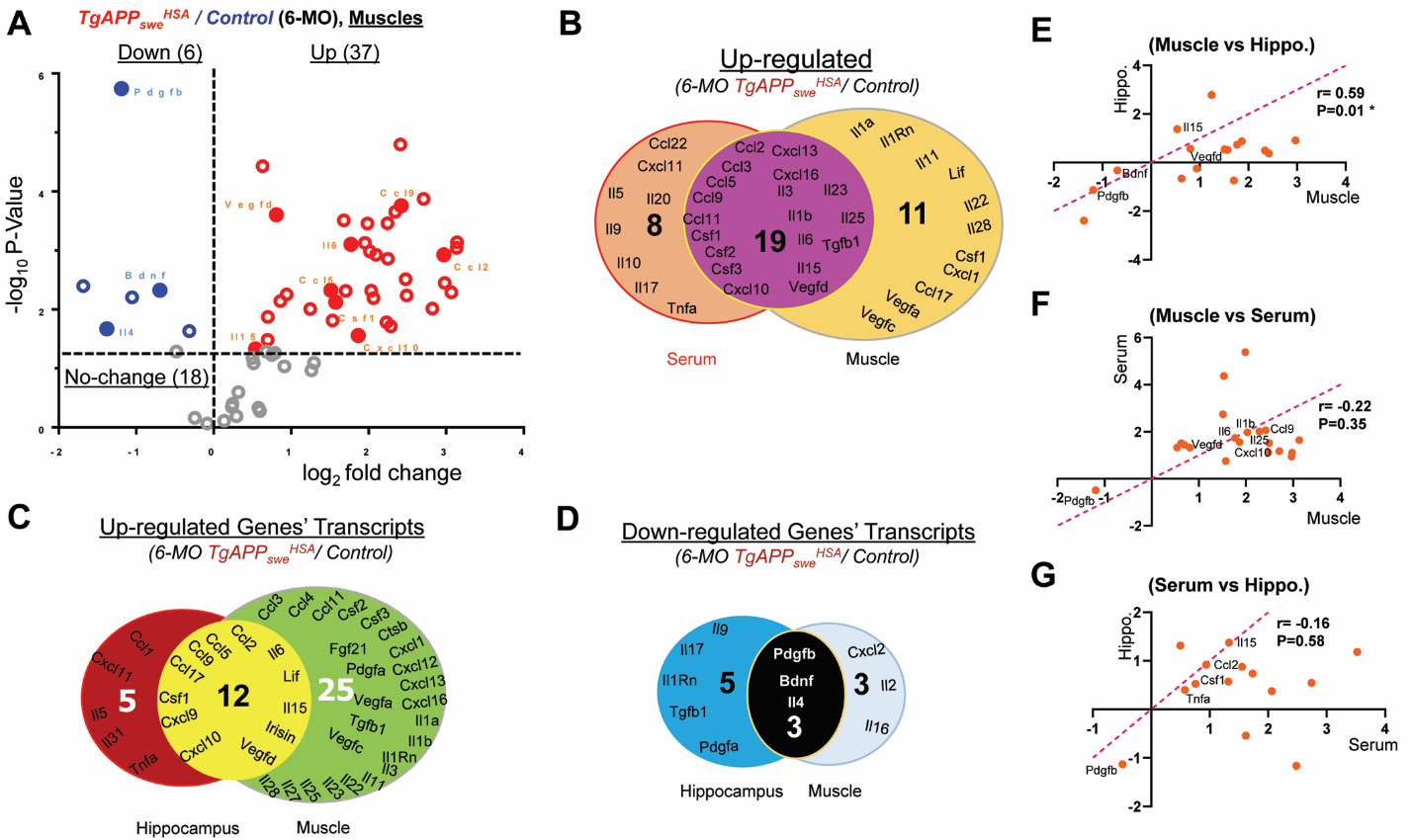


**Supplementary Fig. 11. Increased SASPs in 6-MO *TgAPP_swe_^HSA^* skeletal muscles.** **(A**) Volcano plots analysis of the changes in mRNA levels of 61 genes in 6-MO *TgAPP_swe_^HSA^* TA muscles. *p<0.05 **(B)**Comparison analysis between the serum L-Series label-multiplex antibody array and their mRNA expression in 6-MO TA muscles, 49 factors were tested in both serum and TA muscle samples. Among these 49 factors, 30 factors in serum and 27 factors in muscle were up-regulated in mutant mice, and 19 of them were up-regulated in both mutant serum and muscle samples**.** **(C-D)** Comparison analysis between the mRNA expression (up-regulated in **C** and down-regulated in **D**) of 6-MO TA muscles and hippocampus**. (E-G)** correlation plots of log_2_FC (Fold change) in muscle vs. hippocampus, muscle vs. serum and serum vs. hippocampus for the transcripts significantly regulated in *TgAPP_swe_^HSA^* were shown.


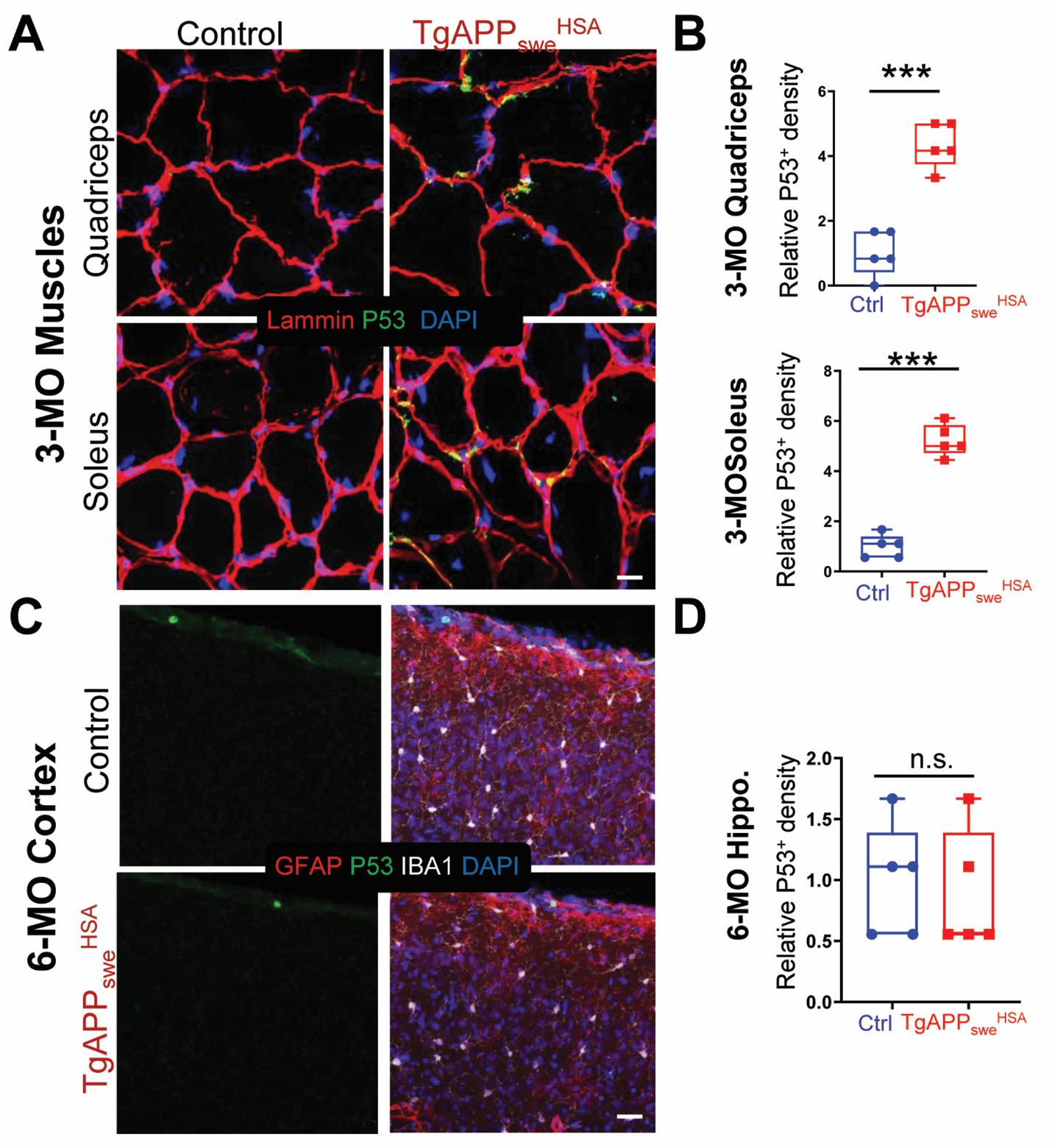


**Supplementary Fig. 12. Increased P53+ cells in the soleus, quadricep muscles but not in the cortex of *TgAPP_swe_^HSA^* mice.** **(A)** Representative images of double immunostaining of laminin (red) and P53 (green) in the quadriceps and soleuses from 3-MO control and *TgAPP_swe_^HSA^* mice. Scale bar, 20*µ*m. **(B)** Quantification of relative P53+ cell density in **A**. **(C)** Representative images of triple immunostaining of GFAP (red), P53 (green) and IBA1(white) in the cortex from 6-MO control and *TgAPP_swe_^HSA^* mice. Scale bar, 20*µ*m. **(D)** Quantification of relative P53+ cell density in **C**. Data are shown as box and whiskers (n=5 mice/group), ***p<0.001, student’s t test.

**
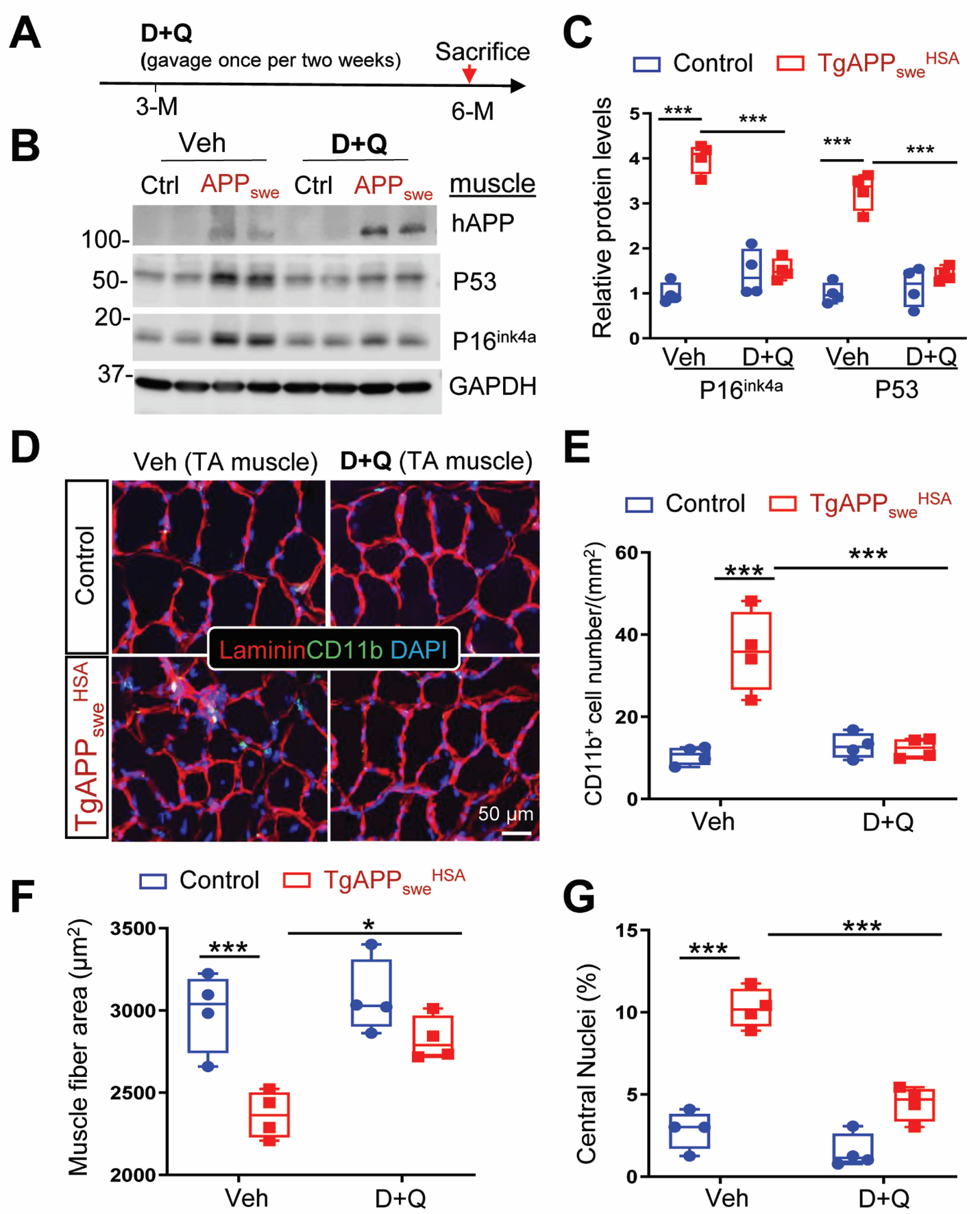
**

**Supplementary Fig. 13. Abolished muscle phenotypes in 6-MO *TgAPP_swe_^HSA^* mice by treatment with senescence inhibitors, D + Q. (A)** Schematic diagram of the experimental design. 6-MO control and *TgAPP_swe_^HSA^* mice were treated with Veh (10%PEG 400) or DQ (D 5mg/kg, Q 50mg/kg, dissolved in 10% PEG 400, once per two weeks), starting at the age of 3-MO, and then subjected to indicated experiments at 6-MO.**(B)** Western blot analysis of indicated protein expression in TA muscles of 6-MO control and *TgAPP_swe_^HSA^* with Veh or DQ treatment. **(C)** Quantification analyses of the data in **B**, data are shown as box and whiskers, ***p<0.001, n=4 mice/group. **(D)** Representative images of immunostaining of CD11b (green) and Laminin (red) with DAPI in TA muscles of indicated genotypes. Scale bar, 50*µ*m. **(E-G**) Quantification of CD11b^+^ cell densities (**E**), mean muscle fiber size (**F**) and central nuclei percentages (**G**) in **D**, data are shown as box and whiskers, n=4, *p<0.05, ***p<0.001. Two-way ANOVA followed by Sidak multiple comparisons post hoc test was used in **C** and **E-G**.


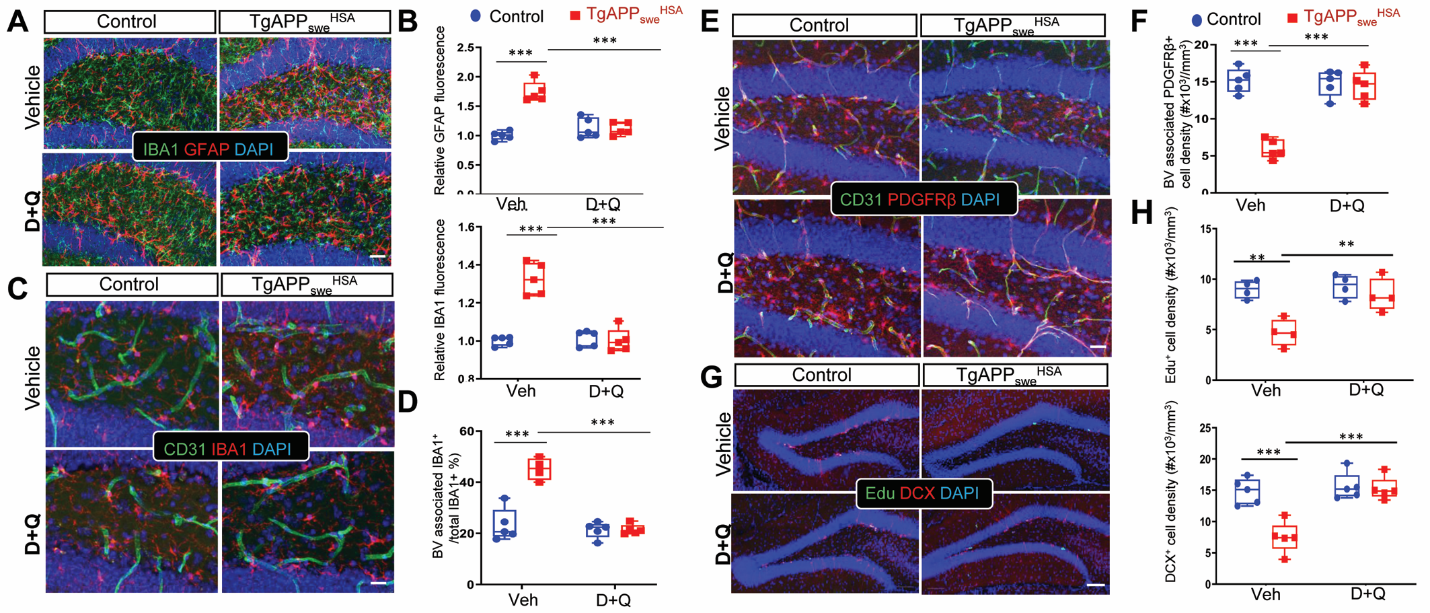


**Supplementary Fig. 14. Attenuated gliosis, pericyte deficit, blood vessel associated microglia and neurogenesis deficit in *TgAPP_swe_^HSA^* mice treated with senescence inhibitor.**

**(A)** Representative images of hippocampal hilus sections from 6-MO control (*LSL-APP_swe_*) and *TgAPP_swe_^HSA^* with Veh or DQ treatments coimmunostained with IBA1 (green), GFAP (red), and DAPI (blue). Scale bar, 20*µ*m. **(B)** Quantification analyses of the GFAP and IBA1 fluorescence intensity of the data in **A**. **(C)** Representative images of hippocampal hilus sections from 6-MO control (*LSL-APP_swe_*) and *TgAPP_swe_^HSA^* with Veh or DQ treatments coimmunostained with CD31 (green), IBA1 (red), and DAPI (blue). Scale bar, 20*µ*m. **(D)** Quantification analyses of the blood vessel-associated microglia in **C. (E)** Representative images of hippocampal hilus sections from 6-MO control (*LSL-APP_swe_*) and *TgAPP_swe_^HSA^* with Veh or DQ treatments coimmunostained with CD31 (green), PDGFRβ(red), and DAPI (blue). Scale bar, 20*µ*m. **(F)** Quantification analyses of the PDGFRβ^+^ cell numbers in **E.** (**G**) Representative images of dorsal DG from 6-MO control and *TgAPP_swe_^HSA^* mice coimmunostained with DCX (Red), Edu(green) and DAPI (blue). Scale bar, 100µm. **(H)** Quantification analyses of Edu^+^ and DCX^+^ cell density in dorsal DG of data in **G**. ****P* < 0.001, n=5 mice/group, the data are presented as box and whiskers, two-way ANOVA analysis followed by Sidak’s multiple comparison post hoc test.

^
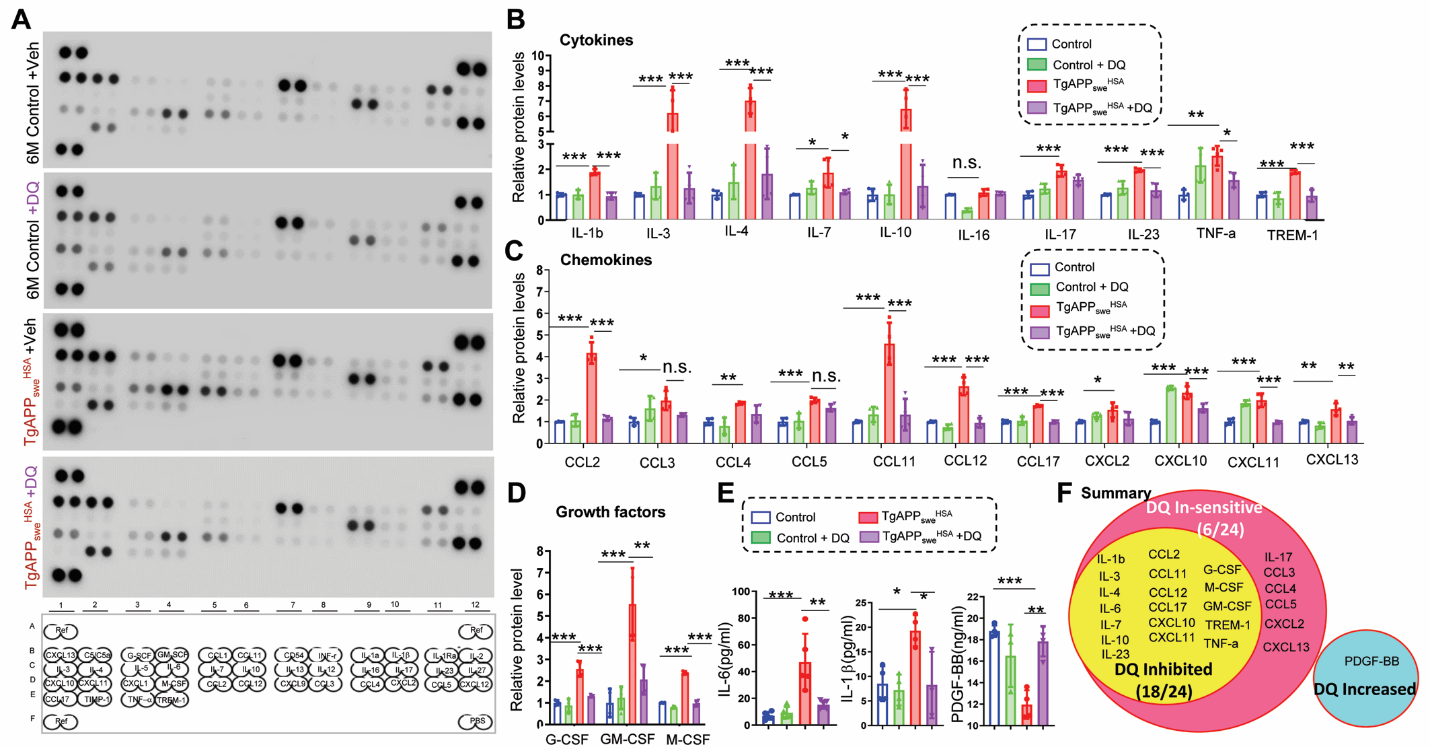
^

**Supplementary Fig. 15. Attenuated SASPs in *TgAPP_swe_^HSA^* mice treated with senescence inhibitors.** **(A**) Proteome profile of Mouse Cytokine Array of 6-MO control and *TgAPP_swe_^HSA^* mouse (male) serum with Veh or DQ treatments. **(B-D)** Quantification analyses of the data in **A**. The values are presented as mean ± SD. *p<0.05, **p<0.01, ***p < 0.001. n=4, two-way ANOVA analysis followed by Sidak’s multiple comparisons test was performed. **(E)** Elisa assays of serum IL6, IL1β and PDGF-BB levels in indicated mice. The data are presented as mean ± SD (n= 3-4 mice). *p<0.05, **p<0.01, ***p < 0.001, two-way ANOVA followed by Tukey ‘s multiple comparisons test. **(F)** Summaries of DQ drugs’ effect on the factors tested in serum.
