# Supplementary material for: Muscular Swedish mutant APP-to-Brain axis in the development of Alzheimer’s disease
Source: Cell Death Dis. 2022 Nov 10;13(11):952. doi: 10.1038/s41419-022-05378-4 (PMC9649614; doi:10.1038/s41419-022-05378-4)
Supplement: Supplementary file 3 — Original western blots [file 41419_2022_5378_MOESM3_ESM.pdf]

Fig. S1D

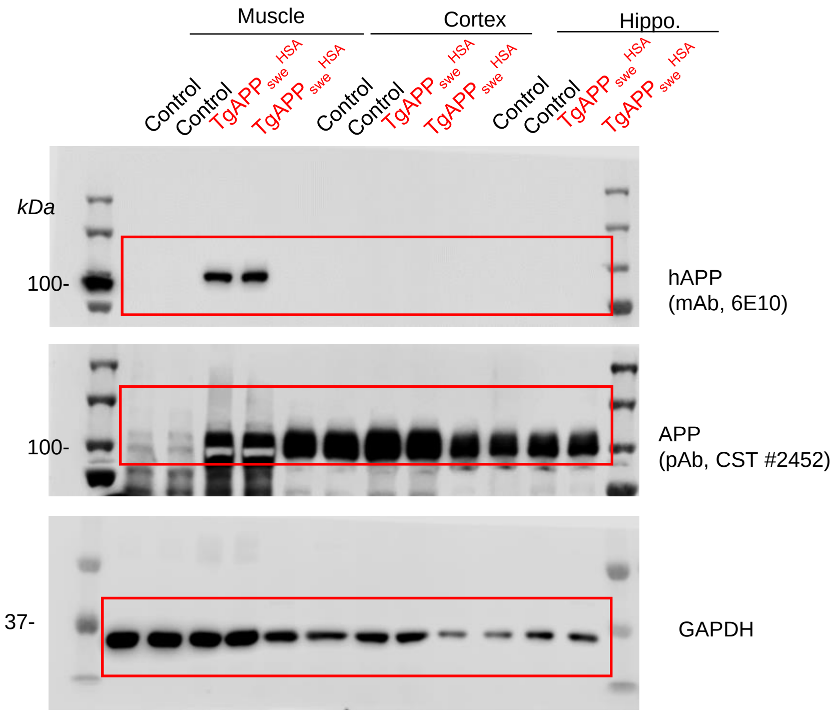
Muscle
Cortex
Hippo.
Control
Control
TgAPP swe HSA
TgAPP swe HSA
Control
Control
TgAPP swe HSA
TgAPP swe HSA
Control
Control
TgAPP swe HSA
TgAPP swe HSA
kDa
100-
hAPP
(mAb, 6E10)
100-
APP
(pAb, CST #2452)
37-
GAPDH

Fig. 2I

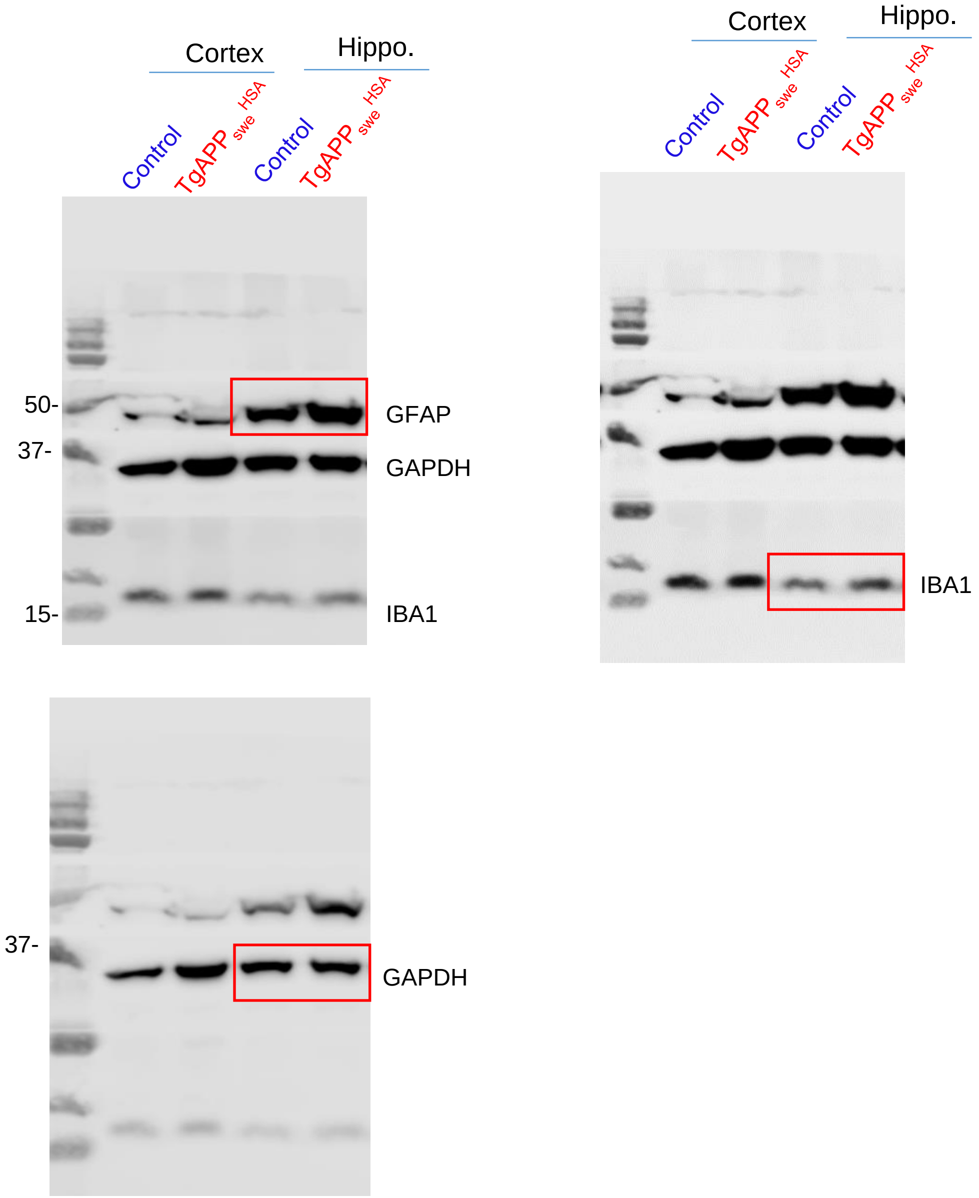

Cortex
Hippo.
Control
TgAPP swe HSA
Control
TgAPP swe HSA
50-
37-
15-
GFAP
GAPDH
IBA1
Cortex
Hippo.
Control
TgAPP swe HSA
Control
TgAPP swe HSA
IBA1
37-
GAPDH

Fig. S5D

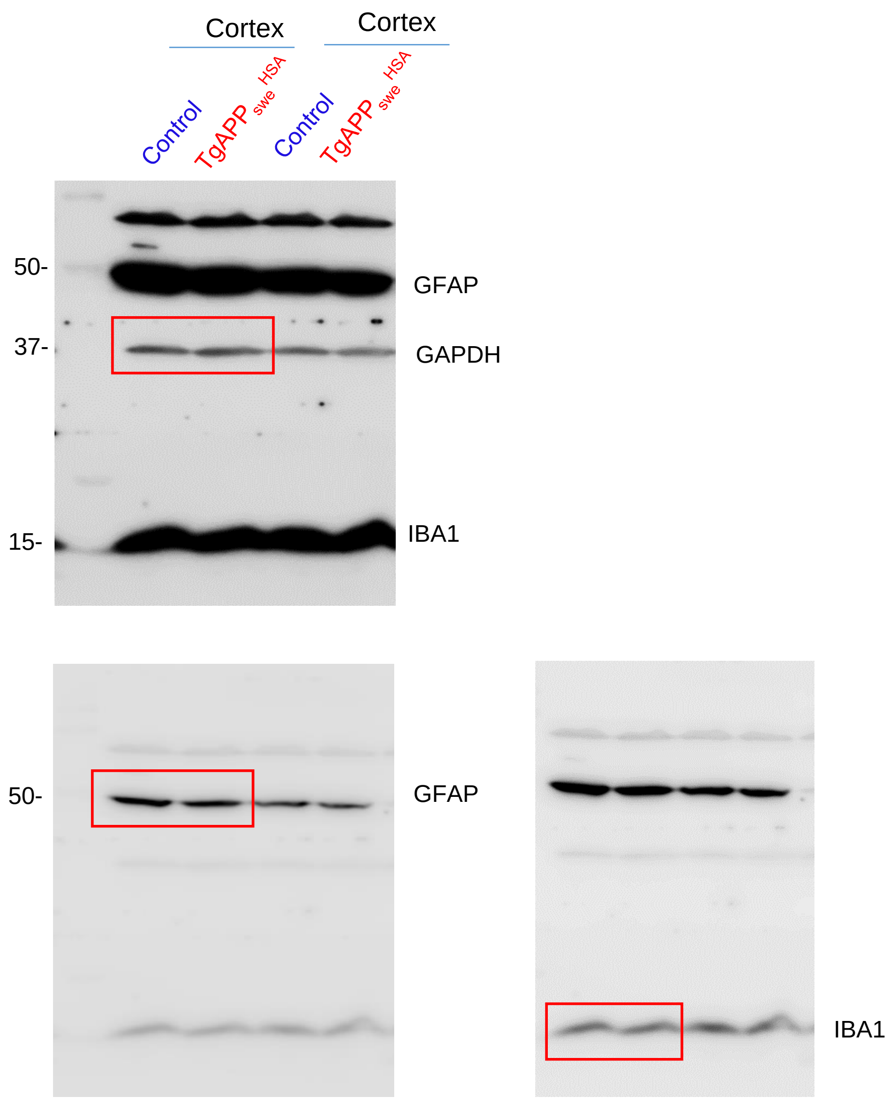
Cortex
Cortex
Control
TgAPP swe HSA
Control
TgAPP swe HSA
50-
37-
15-
GFAP
GAPDH
IBA1
50-
GFAP
IBA1

Fig. 6C

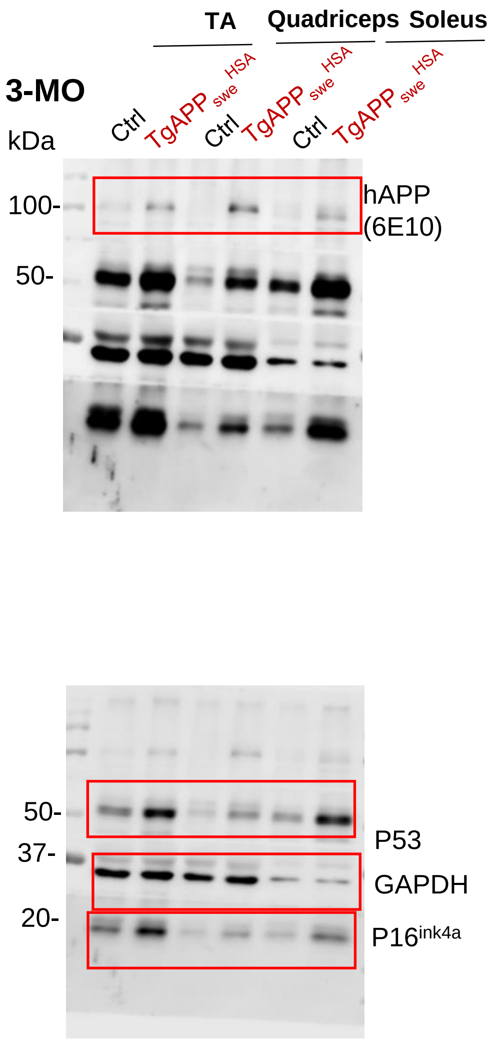
TA
Quadriceps
Soleus
3-MO
kDa
Ctrl
TgAPP swe HSA
100-
50-
hAPP
(6E10)
50-
37-
20-
P53
GAPDH
P16ink4a

Fig. 6J

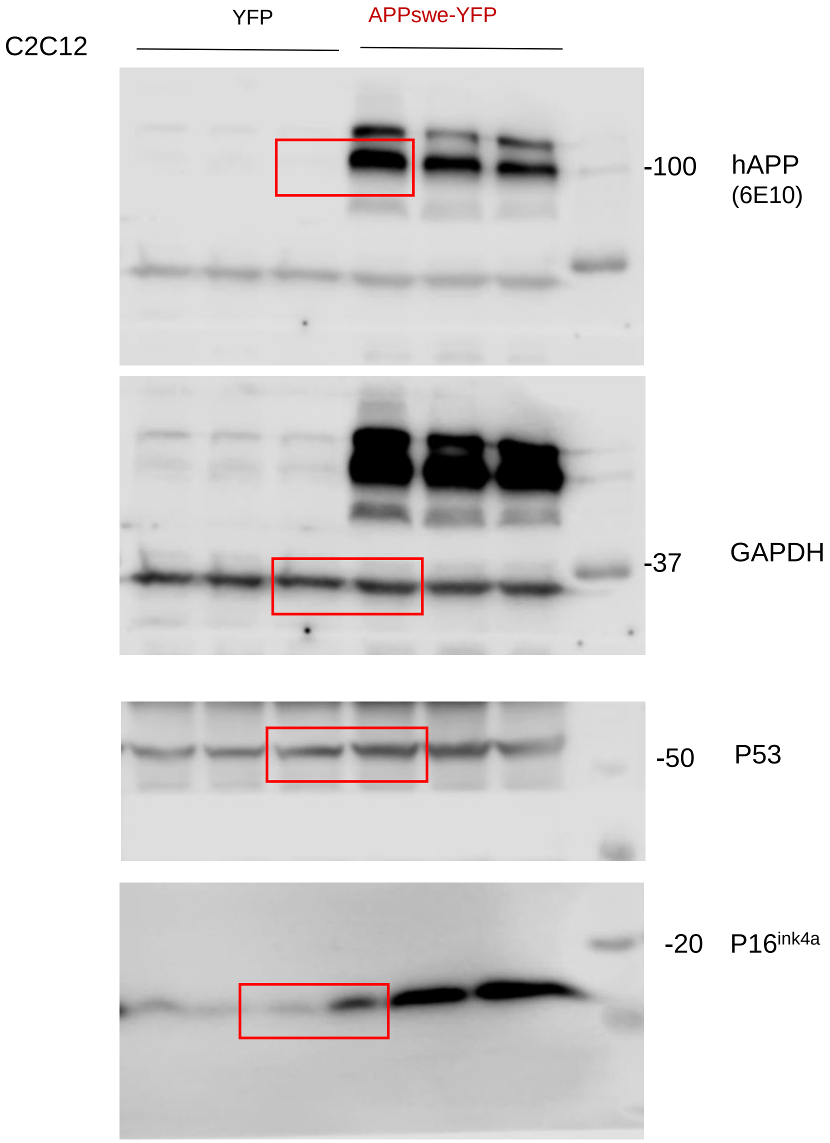
C2C12
YFP
APPswe-YFP
-100
hAPP
(6E10)
-37
GAPDH
-50
P53
-20
P16ink4a

Fig S13B

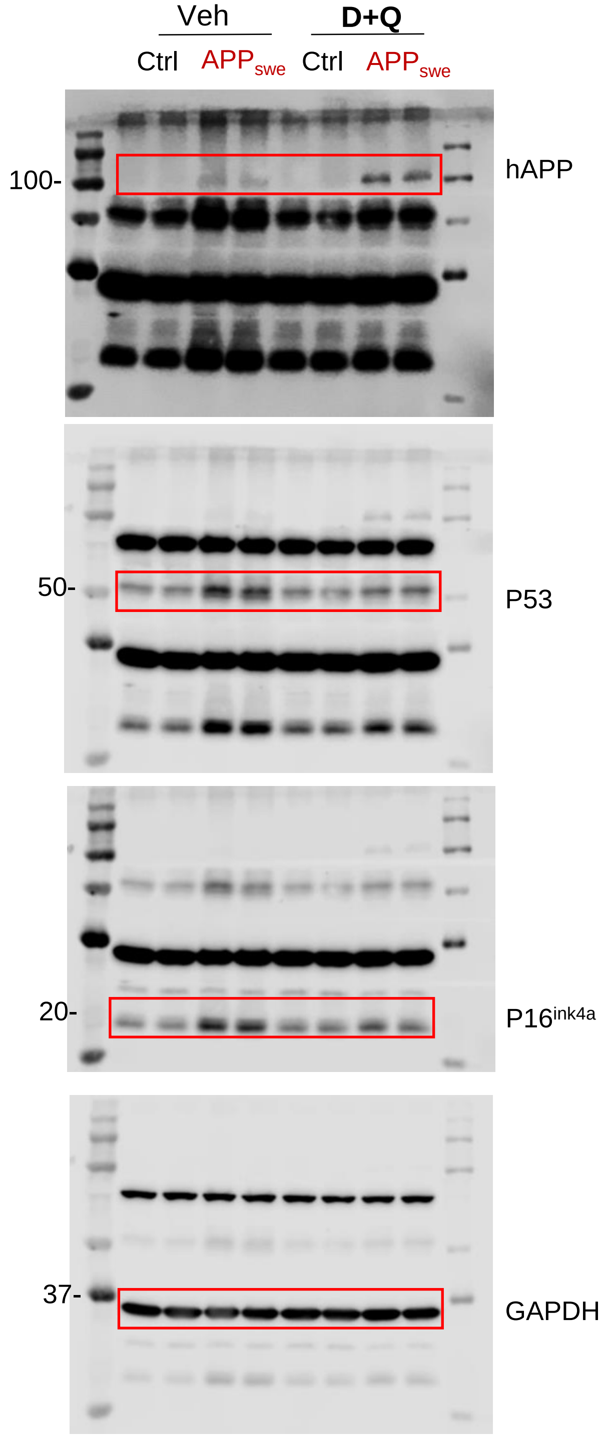
Veh
D+Q
Ctrl
APPswe
Ctrl
APPswe
100-
hAPP
50-
P53
20-
P16ink4a
37-
GAPDH
